# Supplementary material for: Intensive longitudinal follow-up of cisgender and transgender women engaged in sex work during the three months following initiation of daily oral PrEP: A series of case-studies with mixed-method assessments
Source: PLOS Glob Public Health. 2026 May 7;6(5):e0006056. doi: 10.1371/journal.pgph.0006056 (PMC13152121; doi:10.1371/journal.pgph.0006056)
Supplement: S1 Table — (PDF) [file pgph.0006056.s001.pdf]

S1 Table. Summary of descriptive quantitative findings at the individual level

ID	Response rate to daily questionnaire	Days with reported PrEP adherence		Days with reported side effects	Days with reported clients	Mean number of clients per day (min-max)	Days with reported condom use in all encounters*
		Out of all study days	Out of days with answer to daily questionnaire				
1[t]	100% (94/94)	100% (94/94)	100% (94/94)	1% (1/94)	98.9% (93/94)	3.7 (1-7)	86% (87/93)
2[t]	93.2% (83/89)	93.2% (83/89)	100% (83/83)	0	57.3% (51/89)	4 (1-10)	9.8% (5/51)
3[t]	48.3% (45/93)	37.6% (35/93)	77.7% (35/45)	53.3% (24/45)	48.8% (22/45)	2.5 (1-10)	100% (22/22)
4[t]	64.4% (58/90)	64.4% (58/90)	100% (58/58)	50% (29/58)	62% (36/58)	3.1 (1-10)	97.2% (35/36)
5[t]	26.6% (24/90)	24.4% (22/90)	91.6% (22/24)	45.8% (11/24)	37.5% (9/24)	1.3 (1-3)	88.8% (8/9)
6[t]	24.4% (22/90)	24.4% (22/90)	100% (22/22)	36.3% (8/22)	54.5% (12/22)	1.5 (1-6)	75% (9/12)
7[t]	90.1% (82/91)	84.4% (76/91)	92.6% (76/82)	12.2% (10/82)	43.9% (36/82)	2.4 (1-5)	58.3% (21/36)
8[t]	75.5% (68/90)	74.4% (67/90)	98.5% (67/68)	10.2% (7/68)	88.2 % (60/68)	3.7(1-8)	100% (60/60)
9[t]	86.6% (78/90)	76.6% (69/90)	88.4% (69/78)	42.3% (33/78)	44.8% (35/78)	2.3 (1-5)	88% (31/35)
10[t]	83.3% (75/90)	80% (72/90)	96% (72/75)	33.3% (25/75)	78.6% (59/75)	3 (1-6)	9.3% (7/75)
11[t]	82% (74/90)	80% (72/90)	97.2% (72/74)	25.6% (19/74)	62.1% (46/74)	2.5 (1-6)	36.4% (27/74)
12[t]	77.7% (70/90)	73% (66/90)	94.2% (66/70)	7.1% (5/70)	52.8% (37/70)	2.3 (1-5)	35.7% (25/70)
13[c]	97.8% (89/91)	94.5% (86/91)	94.5% (86/91)	65.9% (60/91)	54.9% (50/91)	2.9 (1-5)	100% (91/91)
14[c]	86.3% (76/88)	84% (74/88)	97.3% (74/76)	0	25% (22/88)	2.1 (1-4)	100% (22/22)
15[c]	3% (3/96)	3% (3/96)	100% (3/3)	100% (3/3)	100% (3/3)	3.6 (2-5)	100% (3/3)

*Days in which participant reported having clients, t= transgender woman, c= cisgender woman
