# Supplementary material for: Intensive longitudinal follow-up of cisgender and transgender women engaged in sex work during the three months following initiation of daily oral PrEP: A series of case-studies with mixed-method assessments
Source: PLOS Glob Public Health. 2026 May 7;6(5):e0006056. doi: 10.1371/journal.pgph.0006056 (PMC13152121; doi:10.1371/journal.pgph.0006056)
Supplement: S2 Table — (PDF) [file pgph.0006056.s002.pdf]

S2 Table. Joint display table with individual reports of PrEP use and side effects

ID	Results	
1[t]	**Visual representation**	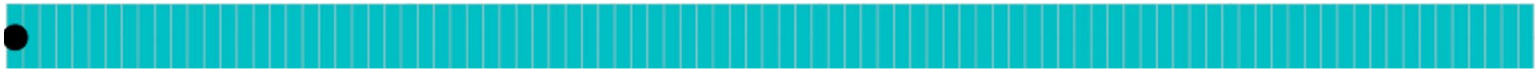
	**Qualitative findings**	Started PrEP because she was worried about HIV exposure from accidental or intentional condom rupture by clients. She learned about PrEP through the NGOs involved in the study. The first day of PrEP intake she reported flu-like symptoms and GI upset. She felt reassured by knowing that side effects go away in the first days or weeks, she had been told so by the study physician, a friend on PrEP, and had heard about it on YouTube. She used the alarm from the daily phone study survey as a reminder before it became part of her daily routine, and always kept the medication by the bedside. She reports not missing any PrEP doses.
2[t]	**Visual representation**	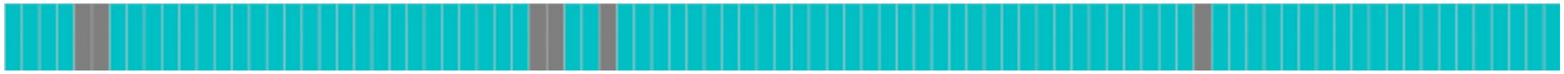
	**Qualitative findings**	She was interested in PrEP due to frequent condomless sex, often for personal pleasure and sometimes for economic reasons, with a small number of selected clients. She learned about PrEP through social media, had immediate interest but discarded the idea to start PrEP when she was told about the long waiting time to be seen by a PrEP provider in Madrid. When she finally started PrEP she was offset about having to take a pill daily, but the expected benefits outweighed those feelings and soon she became used to taking it. She used to take the pill everyday at the same time; however, some times she forgot to take the pill and would end up taking it later the same day. She used to leave the medication everyday in the same place, and would think about taking enough medication with her during trips. Since PrEP is a number one priority for her, she did not needed alarms. She did not miss a single dose of PrEP during the study period. She feels safe taking PrEP, reassured

		she will not acquire HIV, and the intense fear and anxiety that followed condomless sex has disappeared. She advocates about PrEP with other coworkers. She denies side effects from PrEP.
3[t]	**Visual representation**	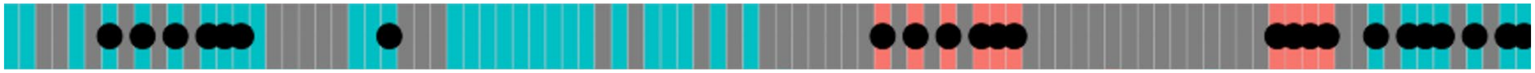
	**Qualitative findings**	She heard about PrEP from the NGO involved in the study. She initiated PrEP due to concerns about intentional condom rupture by clients. She was discouraged to take it by another sex worker who suffered from side effects, but she decided to take it regardless because protecting herself was a priority. She experienced fatigue and nausea soon after starting PrEP, the symptoms lasted approximately one week and she considered stopping PrEP, but continued because she had been told that side effects fade away in days to weeks. She took an stomach protector and paracetamol to help with side effects. She took PrEP along with her daily vitamins, which made it easy to remember. During the study period she was hospitalized for an acute illness (days in orange in the graphic) not related to PrEP and stopped taking the medication. She reported side effects through the daily phone study survey then, but those symptoms were related to her illness. When she resumed PrEP after hospital discharge she denied suffering any side effects, still, symptoms were reported on the daily phone study survey. She reports feels safe since taking PrEP.
4[t]	**Visual representation**	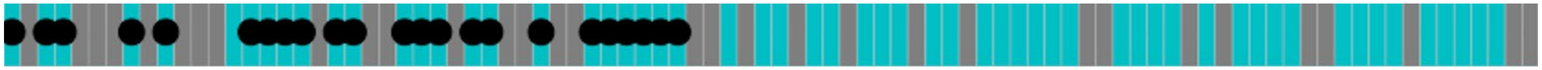
	**Qualitative findings**	She heard about PrEP online though OnlyFans, Grindr and Instagram. She wanted to start PrEP because she felt the pressure to agree to condomless sex to remain competitive with clients, but the fear of acquiring HIV caused her a lot of anxiety. She experienced headache, nausea, reflux and diarrhea after starting PrEP. She shared her experience with a friend that had similar symptoms. To cope with the side effects she started to take her pills after meals, prior to napping, and took an stomach protector for some time. She reported the symptoms as mild and did not think about stopping PrEP because the benefits largely outweighed the side effects. She

		used to leave the pill by the bedside, and did not use alarms or reminders. She did not miss any PrEP doses during the study; however, after the study finished she stopped taking PrEP while being ill and taking antibiotics, as she thought taking the antibiotic and PrEP at the same time would be “too strong” on her stomach.
5t	**Visual representation**	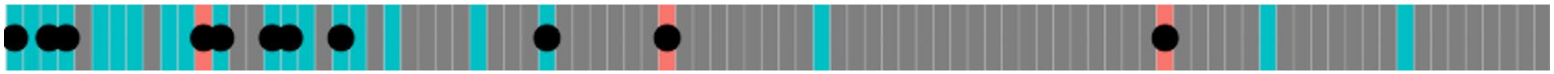
	**Qualitative findings**	She learned about PrEP online. She started PrEP for fear of intentional or accidental condom rupture. After forgetting to take PrEP once, she decided to start using alarms. In addition, she used to keep extra pills and a bottle of water in her purse in case it was time to take PrEP when she was out of her home. There was a time when she stopped taking PrEP due to an elective surgery; however, during that time she was not working. Moreover, she reports suffering from severe cough secondary to asthma/allergies during the study period, which sometimes made her vomit. She did not take the pills in days like those. She thinks that not eating and taking analgesics in addition to PrEP contributed to the nausea and vomiting. These symptoms were reported as side effects of PrEP on the daily phone study survey
6t	**Visual representation**	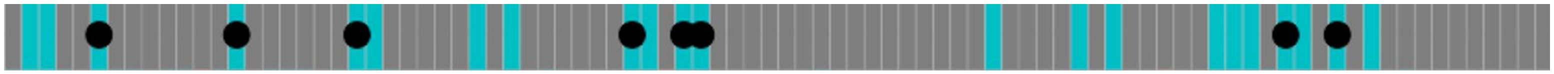
	**Qualitative findings**	She started taking PrEP because she was worried about intentional condom rupture by clients, or unconsented condomless penetration while being intoxicated with drugs or alcohol. She had experience taking post-exposure prophylaxis for HIV in the past after an accidental condom rupture. She heard about PrEP from a friend, who also discouraged her from taking it because of the side effects. Nevertheless, she decided to start PrEP and experienced gastrointestinal upset (nausea, intense burning sensation) and headache. She decided to take the pill every other day, she was not working then, and took paracetamol and a stomach protector to alleviate the symptoms. She considered stopping the pill, but the benefits outweighed the side effects. When she started working a few weeks later she started taking PrEP everyday and took the pill with meals. Symptoms were still

		present but the intensity had decreased. She used first an alarm and then the study survey as a reminder. Part of her routine was to put one pill in her bedside table where she does her make up and replace it everyday. In addition, she used to get a daily phone call from a sibling that would remind her about taking the pill. She missed her pills in two occasions, but this happened after the study ended. Both occasions was travel related, one time she forgot the pills in the check-in baggage and another time she forgot to refill the stash in her purse. She feels more relaxed and calmed since starting PrEP, although the first few weeks she felt slightly anxious due uncertainty about side effects.
7[t]	**Visual representation**	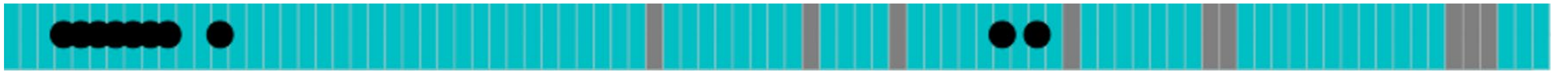
	**Qualitative findings**	She heard about PrEP from coworkers and researched it online. She chose to start taking it out of fear of condom rupture and also to not have to say no to some clients who requested condomless sex. She used to take PrEP along with other medications and used the alarm from the daily phone study survey as a reminder to take the pill, until it became part of her daily routine. Nevertheless, she would occasionally miss the pill 2-3 days in a row. This occurred on the weekends, which is when she used to work and her daily routine changed. She would oversleep due to working many hours or having been under the influence of drugs, and then miss the pill. The first days after starting PrEP she experienced nausea, stomach discomfort and diarrhea. She would have stopped the pill if the symptoms had persisted, but they resolved. She did stop taking PrEP for a few days around Christmas, and again had some side effects after restarting it. She could not recall if the days she did not reply to the daily phone study survey were days in which she took the pill or not.
8[t]	**Visual representation**	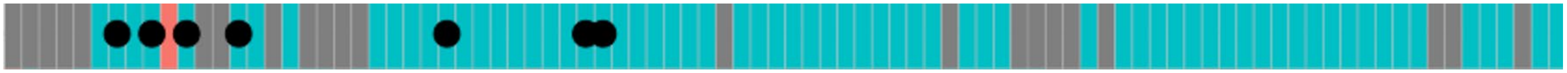
	**Qualitative findings**	She heard about PrEP from the NGO involved in the study and decided to start taking it out of fear of accidental condom rupture, which she had experienced before. She had some mild gastrointestinal upset after starting

		PrEP but it resolved on its own, as she expected. She used to take the pill it everyday after breakfast, the survey alarm reminded her about it. She occasionally missed a dose of PrEP at the usual time due to forgetfulness and ended up taking it later in the day. She missed it one day in Christmas because she has drunk and did not want to mix alcohol and PrEP. She reports the days without answer to the daily phone study survey were days when she took the pill. She thinks she only missed he prep two days during the study period.
**9^{t}**	**Visual representation**	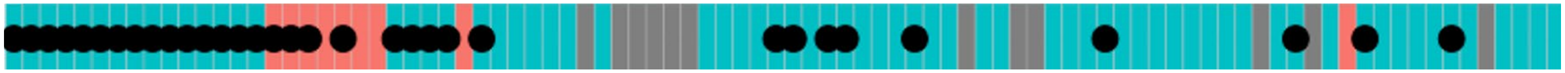
	**Qualitative findings**	No data
**10^{t}**	**Visual representation**	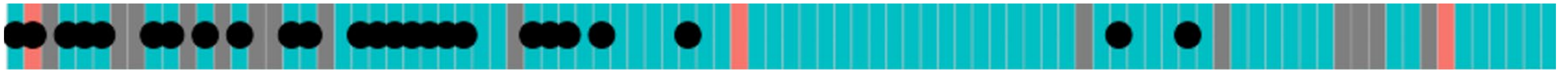
	**Qualitative findings**	She heard about PrEP back in her home country and learned more about it through an NGO involved in the study. She decided to start PrEP to remain healthy for her family who is waiting for her back home. She felt at risk when doing oral sex without condom. She was discouraged to take PrEP by a friend who had experienced diarrhea as a side effect of the medication, but she decided that the benefits were worth the risk. Initially she took PrEP prior to going to bed, to avoid feeling side effects. Then she started taking with the first meal of the day. She used to leave the pill ready by the bedside, not needing any alarms. However, sometimes after working late into the night she would oversleep and forget to take the medication. Because of this she missed the medication a few days, but never two days in a row. She also skipped the pill a few days right at the beginning due to side effects, but after that her compliance improved and the days towards the end of the study period without answer to the daily phone study survey were days in which she reports she took the pill. She suffered nausea and stomach pain after starting PrEP, and then she developed diarrhea, which was bothersome with

		her work. Side effects slowly fed away, as she had expected. She also needed Viagra and wondered if that was a side effect of PrEP.
**11[t]**	**Visual representation**	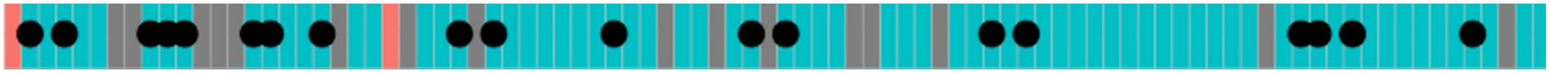
	**Qualitative findings**	She heard about PrEP from the NGO involved in the study and decided to start taking it because she felt exposed to HIV at work and outside work. Sometimes she engaged on condomless sex for economic reasons, sometimes for pleasure, and sometimes it happened as a result of being under the influence of recreational drugs. She also felt a duty to protect her clients who are married, so no illnesses will be transmitted to their families. She was hesitant about starting PrEP due to fear or side effects. Eventually once she started PrEP she experienced mild nausea during the first three months. She attributed it to PrEP and specific circumstances, like eating too little or mixing PrEP with other medications. To cope with the side effects, she would only take PrEP in a full stomach. She missed a few PrEP doses when she had to skip meals due to working long work hours, or when she used recreational drugs at work, to avoid “mixing” them with PrEP. She forgot the pill once while travelling, and she skipped PrEP after the study ended due to an episode of gastroenteritis. She denied nausea or side effects after restarting the medication. She used a phone alarm as a reminder to take the pill, until it became part of her daily routine, as well as a pillbox. She explained that PrEP awoke a strong sense of responsibility, a need to take care of her health, and to stop self medicating and using drugs to shift to a more healthy lifestyle. She reports feeling more responsible since taking PrEP, and feeling at peace about taking care of herself and others since using PrEP.
**12[t]**	**Visual representation**	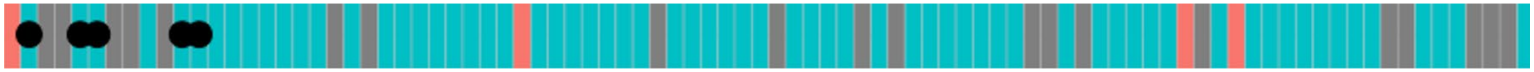
	**Qualitative findings**	No data

13[c]	**Visual representation**	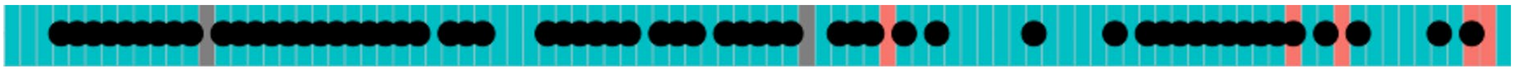
	**Qualitative findings**	She heard about PrEP from the NGO involved in the study and decided to start taking it because of fear of condom rupture, accidental or intentional, even though it had never happened to her because she is very cautious. She experienced nausea and headache as side effects from PrEP. She considered stopping PrEP because the headaches were not improving despite taking medication for it; however, the headaches were mild and she was motivated to continue because she valued more being protected against HIV. It was hard for her to remember to take the pill initially, because she set up to do it at 3 PM, and after a long night of work she would oversleep sometimes. She used to use an alarm and had the pill ready by the bedside with a glass of water. In addition, her sister, also a PrEP user, would remind her to take it. She forgot the pill a couple of days because she wasn't at home when it was time to take it, and by the time she arrived home it was too late and she opted to skip the dose to avoid taking two PrEP doses too close in time. She missed several days of PrEP towards the end of the study because she moved to a different city, and another dose when she visited the emergency department for an acute illness. She reports becoming more responsible since taking PrEP. She also reports stopping drug use since taking PrEP, because the few times she took drugs or alcohol while taking PrEP, she became acutely ill with vomiting. After starting PrEP she continued to pay a lot of attention to condom use, but at least she felt at ease.
14[c]	**Visual representation**	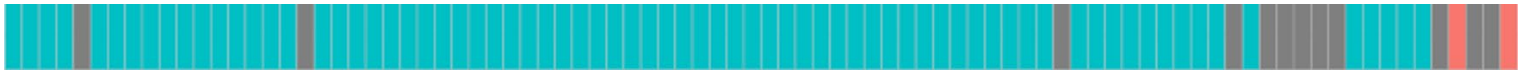
	**Qualitative findings**	She heard about PrEP from the NGO involved in the study and decided to start taking it because she felt at risk of HIV due to agreeing to do oral sex without condom in order to remain competitive. She had no side effects from PrEP. She kept it locked and remembered to take it everyday at the same time without need of alarms. She stopped PrEP towards the end of the study period because she stopped being a sex worker.

**15[c]**	**Visual representation**	No data
	**Qualitative findings**	She learned about PrEP from an NGO involved in the study and decided to start PrEP because she was afraid of contracting HIV from condomless oral sex or due to condom rupture. She found hard to remember to take the pill initially, and she was discouraged about the size of the pill. In addition, she suffered from nausea and malaise the first 15 days and even thought about stopping the pill, but had the support of other coworkers on PrEP who went through a similar experience. Eventually the symptoms disappeared and PrEP became part of her daily routine. She used to take the pill around two in the afternoon, and used to use an alarm as a reminder. Despite this, she would forget sometimes. Overall she reports taking more than half of the pills prescribed to her. She did stop the PrEP at the time of the interview when she was diagnosed with a urinary tract infection, but was planning to resume it in the future.

Blue= taken, orange= not taken, dot= reported side effect, gray= missing data, t= transgender woman, c= cisgender woman.
