# Supplementary material for: Intensive longitudinal follow-up of cisgender and transgender women engaged in sex work during the three months following initiation of daily oral PrEP: A series of case-studies with mixed-method assessments
Source: PLOS Glob Public Health. 2026 May 7;6(5):e0006056. doi: 10.1371/journal.pgph.0006056 (PMC13152121; doi:10.1371/journal.pgph.0006056)
Supplement: S3 Table — (PDF) [file pgph.0006056.s003.pdf]

S3 Table. Joint display table with individual reports of condom use

ID	Results	
1[t]	Visual representation	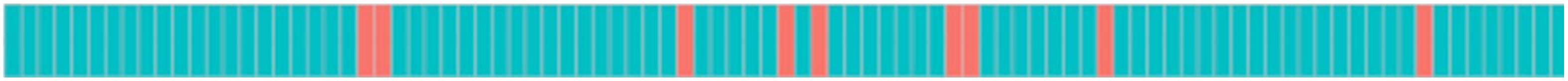
	Qualitative findings	She reports that she continued using condoms more than 90% of the time. She disclosed PrEP use to few clients and a “boyfriend” with whom she wanted to have condomless sex for personal pleasure. She reports it was also a way to “try out” or verify the effectiveness of the medications. She continued using condoms most of the time because she is afraid of getting sexually transmitted infections other than HIV. Additionally, she feels protected against HIV and is not as worried as she used to be about checking the condom during sex.
2[t]	Visual representation	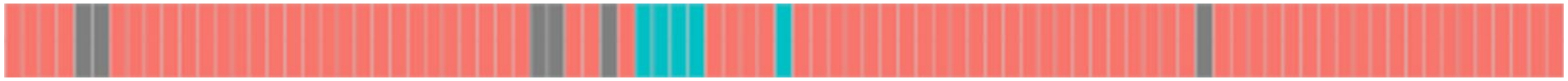
	Qualitative findings	She reports she engaged in condomless sex for personal pleasure with “attractive clients” or for economic reasons, with a minority of clients. She acknowledges these clients requested her services more often since being on PrEP. With the rest of her clients she remained very strict with regards to condom use.
3[t]	Visual representation	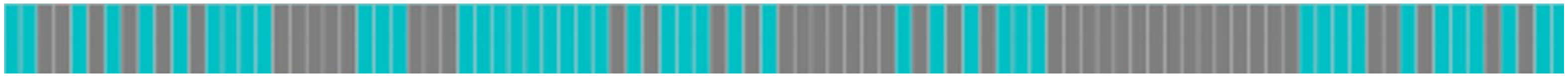
	Qualitative findings	She reports she was tempted to engage in condomless sex when facing economic difficulties. However, she continued using condom with all clients, as she heard PrEP is not 100% effective and needs to be used alongside condoms. She is also afraid of acquiring other sexually transmitted infections though condomless sex.

4t	**Visual representation**	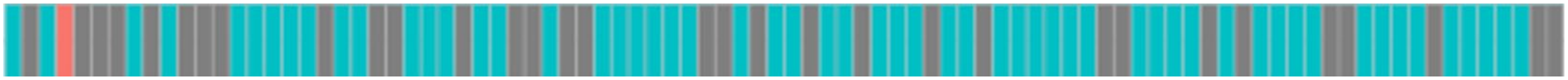
	**Qualitative findings**	She explains that she used to have condomless sex with a few loyal long term clients prior to PrEP. After starting PrEP she became more relaxed about condom use and agreed to do oral sex without condom to handsome clients offering extra money, which is a service she did not use to provide before PrEP. She did have one condomless encounter with penetration soon after initiating PrEP with one of her long standing clients. She shared information about PrEP intake with clients.
5t	**Visual representation**	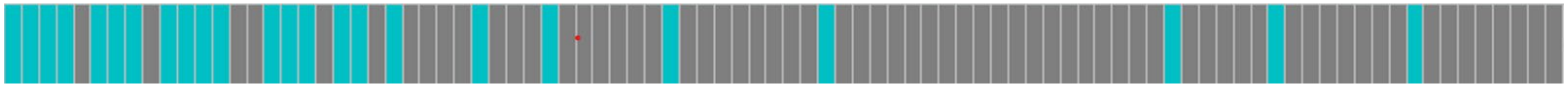
	**Qualitative findings**	She explains that prior to PrEP she used to use double condom and always said no to oral sex. She reports that she continued to follow the same precautions after starting PrEP.
6t	**Visual representation**	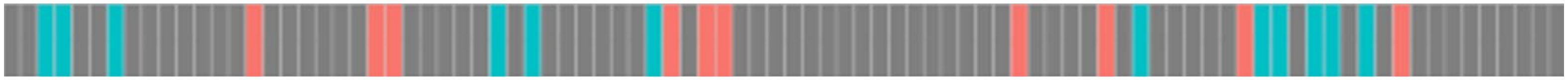
	**Qualitative findings**	She reports she started doing more oral sex and rubbing of the genitals without condom after starting PrEP. Contrary to what is reported on the daily phone study survey, she denies penetration without condom after starting PrEP.
7t	**Visual representation**	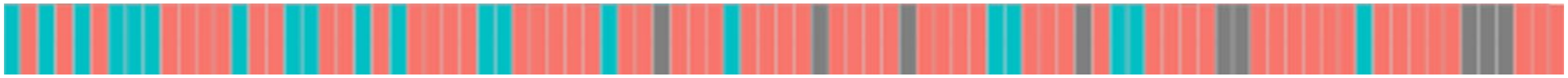

	**Qualitative findings**	She reports that she continued to have condomless encounters with one or two loyal clients with whom she used to have unprotected sex before PrEP. In addition, while on PrEP she started to occasionally agree to having condomless sex with non-loyal clients when she was in economic need.
**8t**	**Visual representation**	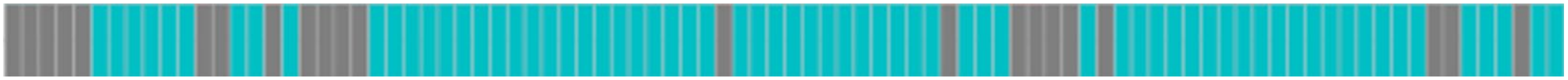
	**Qualitative findings**	She continued to protect herself as much as she used to before taking PrEP, as she was afraid of other sexually transmitted infections.
**9t**	**Visual representation**	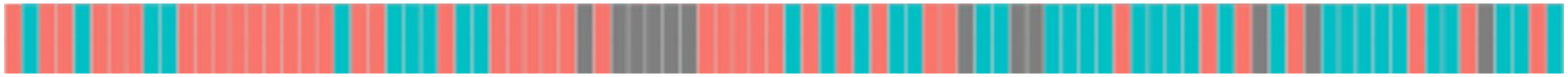
	**Qualitative findings**	No data
**10t**	**Visual representation**	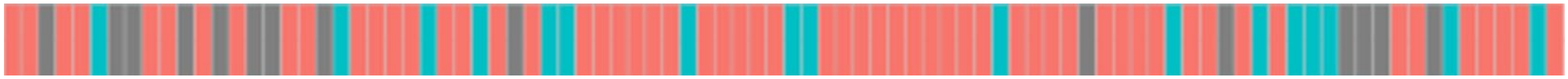
	**Qualitative findings**	She reports a drop of approximately 10% in condom use after starting PrEP. She reports work had been difficult and as a consequence she stopped using condoms with some clients, not for pleasure but because of economic need. She gives the example of loyal clients and clients with allergy to latex. She did feel safer being on PrEP, and did not regard other sexually transmitted infections as being as bad as HIV. She also acknowledged condomless sex with one partner.

11[t]	**Visual representation**	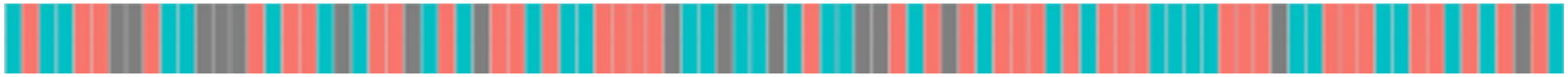
	**Qualitative findings**	She reports she used condoms even more than before. She explains that the number of clients decreased due to policy changes around sex trade, but the clients remaining were more responsible. She reports she marked as days not using condom in the daily phone study survey days in which she had clients who want masturbation or just conversation.
12[t]	**Visual representation**	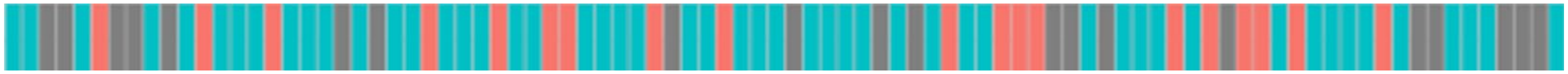
	**Qualitative findings**	No data
13[c]	**Visual representation**	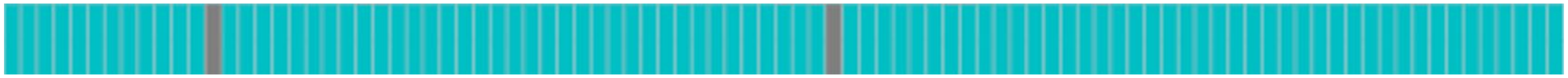
	**Qualitative findings**	She reports nothing changed with regards to condom use, she continued to be very vigilant and use condoms in all encounters. However, she reports she had several incidents in which the condom accidentally ruptured after starting PrEP, but she did not worry about it because she was being complaint with PrEP and after the episode she did not show signs or symptoms of illness.
14[c]	**Visual representation**	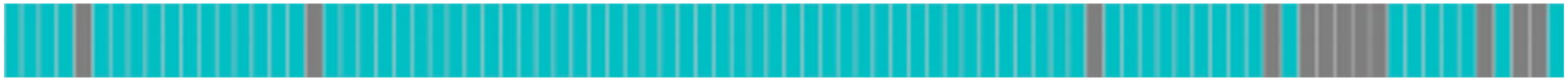

	**Qualitative findings**	She reports she continued using condoms as usual after starting PrEP, amongst other things, because she was not sure of how effective it will be. She also had clients with family etc, that were interested in condom use.
**15[c]**	**Visual representation**	No data
	**Qualitative findings**	She reports she continued to use condoms with all clients after initiating PrEP.

Blue= used condom in all sexual encounters that day or had no clients, orange= condom use in less than 100% of sexual encounters that day, grey= no answer,t= transgender woman, c= cisgender woman
