# Supplementary material for: Intensive longitudinal follow-up of cisgender and transgender women engaged in sex work during the three months following initiation of daily oral PrEP: A series of case-studies with mixed-method assessments
Source: PLOS Glob Public Health. 2026 May 7;6(5):e0006056. doi: 10.1371/journal.pgph.0006056 (PMC13152121; doi:10.1371/journal.pgph.0006056)
Supplement: S4 Table — (PDF) [file pgph.0006056.s004.pdf]

S4 Table. Joint display table with individual reports of number of clients

ID	Visual representation	Qualitative findings
1[t]	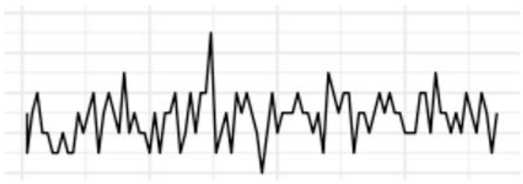	Her relationship with clients remained the same after starting PrEP, with the exception that she disclosed PrEP use to “two or three” clients and a “boyfriend” with whom she wanted to have condomless sex. She denies a relation between overall number of clients and PrEP use.
2[t]	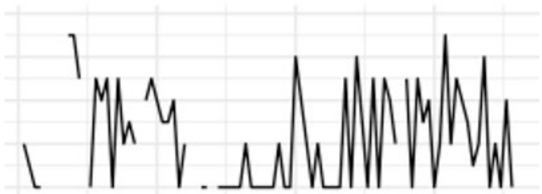	She disclosed PrEP use to clients whom she knows for several years and who are loyal clients, as a way to strengthen their relationship. She also had relationships with non-clients during this time. She said it was a possibility that she had more clients than usual after initiating PrEP because she was more agreeable to condomless sex. The period in which the number of clients drop in the graphic corresponds to a trip back to her home country.
3[t]	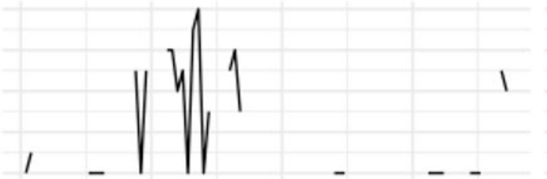	She denies a relation between overall number of clients and PrEP use. She did not disclose PrEP intake to clients. The change in number of clients had to do with period of illness and other work related reasons.
4[t]	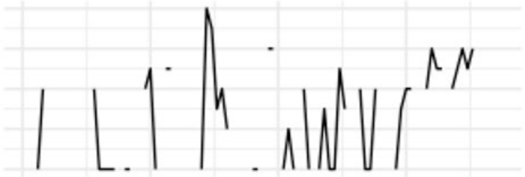	She denies a relationship between PrEP use and number of clients, because she even though she talks about PrEP use with clients, she does not agree to condomless penetration, only to condomless oral sex. She thinks if she did, she would have many more clients.

5[t]	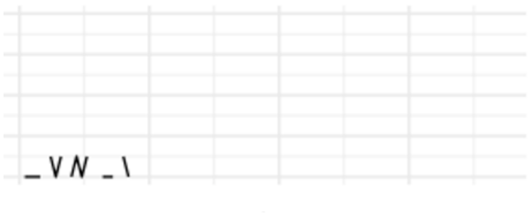	She denies a relationship between PrEP use and the number of clients. She does share information about PrEP with a small number of clients who ask about her health status, along with results of testing for sexually transmitted infections.
6[t]	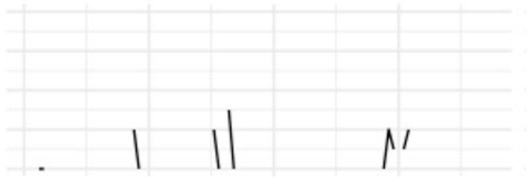	She acknowledges she had more clients after initiating PrEP because she became agreeable to condomless oral sex. She disclosed PrEP use to some clients who asked about the change in condom use.
7[t]	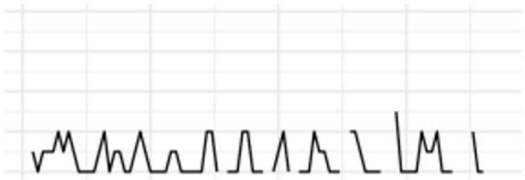	She reports she works on the weekends mostly and denies relationship between PrEP and number of clients.
8[t]	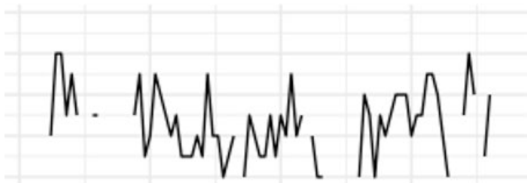	She denies sharing information about PrEP use with clients and does not see a relationship between PrEP use and number of clients.
9[t]	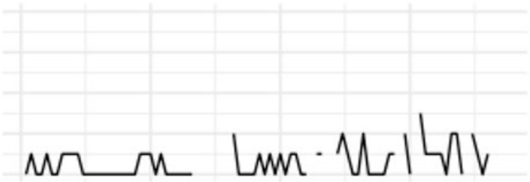	NA

**10[t]**	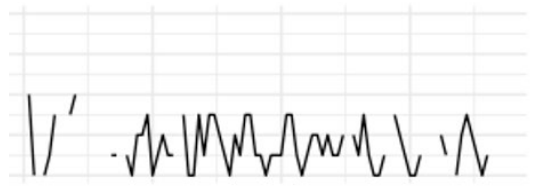	She reports an increase in the number of clients after starting PrEP, which she attributed to a decrease in condom use, which went down to approximately 10%. She shared information about PrEP intake with clients, who she reports had a positive attitude about it.
**11[t]**	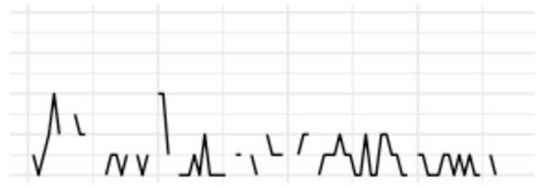	She reports feeling more responsible towards her clients since taking prep, like she is also protecting them. She does not see a relationship between prep and number of clients, since she does not advertise herself as using PrEP on her advertisement. She has been asked about PrEP by some clients, she explains she uses it as an addition to condom use to protect herself.
**12[t]**	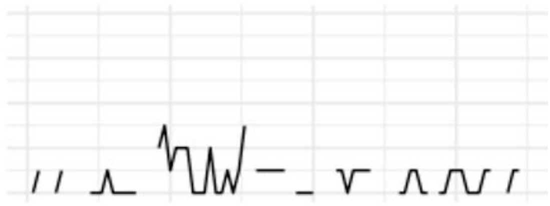	NA
**13[c]**	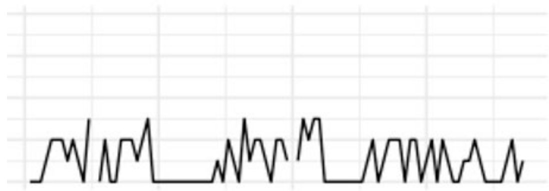	Did not share information about PrEP use with clients because she anticipated request for condomless sex. Denies an impact of PrEP on number of clients, which she correlates with working independently or in an apartment with other coworkers.
**14[c]**	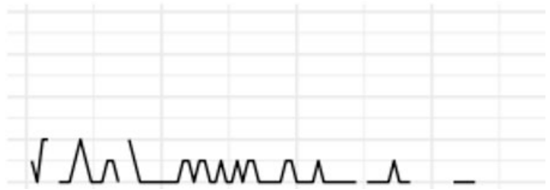	She did not share PrEP use with any client, she denies changes in her relationship with clients since starting PrEP. Does not see a relationship between prep use and number of clients.

**15[c]**	NA	She denies sharing information about PrEP use with clients and does not see a relationship between PrEP use and number of clients.

Scale for number of clients per day goes from 0 to 8 on the Y axis, t= transgender woman, c= cisgender woman
