# Supplementary material for: Intensive longitudinal follow-up of cisgender and transgender women engaged in sex work during the three months following initiation of daily oral PrEP: A series of case-studies with mixed-method assessments
Source: PLOS Glob Public Health. 2026 May 7;6(5):e0006056. doi: 10.1371/journal.pgph.0006056 (PMC13152121; doi:10.1371/journal.pgph.0006056)
Supplement: S5 Table — (PDF) [file pgph.0006056.s005.pdf]

S5 Table. Mixed analysis table for PrEP adherence, condom use and number of clients at the individual level

**ID1[t]**	**PrEP adherence:** ID1 had a response rate of 100%. She reported perfect adherence to PrEP on the daily phone study survey, with just one day of side effects. She benefited from the use of alarms, and the creation of a daily routine. PrEP adherence was likely facilitated by having just one day or side effects, and being very well informed about PrEP from different sources (health care staff, internet, coworkers).<br><br>**Condom use:** Through the daily phone study survey she reported not using condom during intercourse 14% of the days in which she had clients. She acknowledged in the interview that condom use for intercourse decreased from 100 to approximately 90% after initiating PrEP. She had condomless sex with selected clients for personal pleasure and only disclosed PrEP use to them. Her main reason to continue using condoms with most clients was fear of other STIs.<br><br>**Number of clients:** The mean number of clients remained stable during the study period, with her denying any relationship between PrEP use and number of clients.
**ID2[t]**	**PrEP adherence:** ID2 had a response rate of 93.2%. Her reported adherence to PrEP based on response to the daily phone study survey was 100%. This is consistent with her interview answers, when she reported excellent adherence and that on the days without answers she also took the medication. She created a daily routine to take PrEP and did not use alarms, she reports it was not necessary because she was very motivated. She did not experience any side effects, which likely contributed to her excellent adherence.<br><br>**Condom use:** Through the daily phone study survey she reported that most days in which she had clients, she had at least one encounter in which she did not use condom for intercourse (90.2%). In the interview she described choosing to do so for pleasure, with long standing loyal clients or with attractive clients.

	**Number of clients:** The results of the daily phone survey appear to show an increase in the number of clients as the study goes on. She acknowledged the possibility that her number of clients had increased after initiating PrEP because she was more agreeable to condomless sex.
**ID3[t]**	**PrEP adherence:** ID 3 had a response rate of 48.3% and a reported adherence of 77.7%. Through the daily phone study survey she reported a period of non adherence which finished before the end of the study, as well as side effects from PrEP throughout the study. During her interview she explains that she transiently stopped taking PrEP due to an acute illness that required hospitalization. At that time of the hospital admission the symptoms reported as side effects through the study survey were actually symptoms from her illness. She did experience nausea and fatigue soon after starting PrEP, but it resolved with the help of paracetamol and a stomach protector pill. Adherence was facilitated by creation of a daily routine and being used to taking other daily medications.<br><br>**Condom use:** The days in which she answered the survey she reported using condoms with all her clients. In the interview she explained that she continued using condoms with all clients even though she was tempted not to for economic reasons. She continued using condoms because she was afraid of acquiring other sexually transmitted infections and PrEP not being 100% effective.<br><br>**Number of clients:** The small number of responses makes it difficult to evaluate the trend on her number of clients. In the interview she denied a relationship between PrEP use and number of clients, since she did not disclose its use to any clients. She had frequent illness during the study period which negatively impacted her work.
**ID4[t]**	**PrEP adherence:** ID 4 had a response rate of 64.4%. She reported taking PrEP all the days in which she answered the survey, and during the first month she reported having side effects. This is consistent with her interview, in which she reported excellent PrEP adherence and suffering mild GI upset that resolved overtime. She benefited from taking the pill prior to her meals and using a stomach protector.

	**Condom use:** She reported one day with condomless intercourse during the study period. In the interview she explained that this took place with a long standing client. Otherwise she decreased the use of condoms in oral sex with clients who were handsome or paid well.<br><br>**Number of clients:** The results of the daily phone survey show a stable number of clients throughout the study. In the interview the participant denies a relationship between the number of clients and PrEP use because even though she talks about PrEP with clients, she only agrees to condomless oral sex.
**ID5**[t]	**PrEP adherence:** ID 5 had a response rate of 26.6%. She reported using PrEP most of the days in which she replied to the survey (91.6%), and having side effects in (45.8%) of those days, even late in the study period. During the interview she reported good adherence, except for when she skipped PrEP due to an scheduled surgical intervention (not reported through the daily surveys), and some scattered days in which she had severe cough leading to vomiting due to seasonal allergies. Some days in which she reported PrEP side effects on the daily phone study survey, she actually was referring to symptoms caused by her allergies. She benefited from using alarm an alarm and keeping extra pills in her purse.<br><br>**Condom use:** She did not report any condomless sexual encounter through the daily phone survey. She reported in the interview that she continued using condoms as she used to prior to taking PrEP.<br><br>**Number of clients:** The scarce data does not allow to visualize a trend.
**ID6**[t]	**PrEP adherence:** ID 6 had a response rate of 24.4%. She reported using PrEP all days in which she replied to the survey. She also reported PrEP related side effects in 36.3% of those days, even late in the study period. During the interview she explained that a few days after starting PrEP she developed GI upset, which got better with time but did not resolve. She also explained that early on she intentionally skipped the medication every other day because of the side effects, she was not working then. She benefited from having a daily routine with an alarm and also a sibling checking on her adherence to PrEP.

	**Condom use:** She reports she started doing more oral sex and rubbing of the genitals without condom after starting PrEP. Contrary to what is reported on the daily phone study survey, she denies penetration without condom after starting PrEP.<br><br>**Number of clients:** The trend appears stable throughout the study, however this result is limited by due to her agreeance to condomless to oral sex.
**ID7ᵗ**	**PrEP adherence:** ID had a response rate of 90.1%. On the days in which she replied to the daily survey her reported adherence to PrEP was 100%. However, during the interview she acknowledged missing PrEP doses 2 to 3 days in a row when she was working, which was usually the weekend, and a few days around Christmas. This probably corresponds to the days without answer to the daily survey, but the participant could not confirm that. She also reported GI upset soon after initiating PrEP that self resolved after a few days and occurred again when she restarted the medication after the Christmas break. This appears reflected on the daily phone study survey results. Both times she had taken the pill for a few days before the side effects took place.<br><br>**Condom use:** She reported not using condoms during intercourse in the 41.7% of study days in which she had clients and she replied to the survey. Based on the answers during the interview, there appears to be an increase in condomless sex after initiating PrEP. She reported that in addition to having condomless encounters with one or two loyal clients, while on PrEP she started to occasionally agree to having condomless sex with non-loyal clients when in economic need.<br><br>**Number of clients:** The trend appears stable on the results from the daily phone study survey, and the participant reports no relationship between prep use and number of clients. Even though she reported an increase in condomless sex, this might not have had a significant repercussion in the overall number of clients overtime.
**ID8ᵗ**	**PrEP adherence:** ID had a response rate of 75.5%. The days in which she replied to the daily survey, she only report missing PrEP one day in the first couple of weeks. During the interview she reported missing PrEP only 2 days during the study period, one of them because she had drunk alcohol and did not want to mix it with PrEP. She had mild GI upset soon after initiating

	PrEP but it self resolved. She benefited from daily alarms, however despite this she would often forget and end up taking the medication later the same day.<br><br>**Condom use:** She reported consistent condom use through the daily survey. She continued to protect herself as much as she used to before taking PrEP, mostly because she was afraid of other sexually transmitted infections.<br><br>**Number of clients:** Trend appears stable on the results from the daily phone study survey and participant reports no relationship between prep use and number of clients.
**ID9[t]**	For ID 9 we have no interview
**ID10[t]**	**PrEP adherence:** ID 10 had a response rate of 83.3%. She took PrEP almost all the days in which she replied to the daily survey (96%) with some missed doses scattered throughout the study period. In the interview she reports that early on she skipped the medication due to side effects (some of this days she did not reply to the daily phone study survey), and later on in the study she missed it because she overslept after working long hours. She denies missing the medication more than one day in a row. Her adherence improved towards the end of the study. She reported having side effects XX% of the days in which she answered the survey. These were distributed throughout the study period. During the interview she explained that she suffered gastrointestinal side effects from PrEP that slowly fed away, as well as impotence leading to the use of Viagra. She was unsure if this was a side effect from PrEP; but since it was new she reported it as such. She benefited from having a daily routine and taking PrEP with meals.<br><br>**Condom use:** She reported not using condoms during intercourse in 88% of the days in which she had clients and replied to the daily phone study survey. This appears to be an increase from her baseline, as she explains in the interview that she went through a difficult time at work and she benefited from PrEP use and condomless sex for economic reasons.<br><br>**Number of clients:** The trend from the results of the daily phone study survey appears to show a decrease in the number of clients, at least early on, however participant reports an increase in the number of clients after starting PrEP, which she attributed to a decrease in condom use, which went down to approximately 10%*.

**ID11t**	**PrEP adherence:** ID had a response rate of 82%. She took PrEP almost all the days in which she replied to the daily survey (97.2%). She missed a few PrEP doses when she had to skip meals due to working long hours, or when she used recreational drugs at work, to avoid “mixing” them with PrEP. She also forgot the pill once while travelling. It is likely that some of the days in which did not answer the daily phone study survey she did not take the pill. She experienced side effects throughout the study period (25.6% of the days with answers to they daily phone study survey), as this was triggered by her not eating well or mixing PrEP with other medicines. She benefited from using an alarm, a pill box, taking the pill with meals and creating a daily routine.<br><br>**Condom use:** She reported condomless sex in 73.6% of the days in which she had clients and she answered the daily phone study survey. However, during the interview she explained that she marked as days without consistent condom use, days in which she did not have intercourse with clients (days in which there was masturbation only, or just conversation).<br><br>**Number of clients:** The trend from the results of the daily phone study survey appears to show a decrease in the number of clients trend appears to go down. Participant did not see a relationship between PrEP and the number of clients. She though it might be different if she mentioned PrEP in her advertisement.
**ID12t**	For ID 14 we have no interview.
**ID13c**	**PrEP adherence:** ID 12 had a response rate of 97.8%. She took PrEP almost all the days in which she replied to the daily survey (94.5%), with decrease adherence towards the end of the study. In the interview she explained that she missed the pill a couple of days because (1) she wasn’t at home when it was time to take it, (2) she visited the emergency department, and (3) towards the end of the study period she moved to a different city. She suffered from mild headache throughout the study (65.9% of the days in which she answered the survey), which did not improved with paracetamol but it was mild and she decided to continue PrEP despite it. She benefited from having a daily routine and using a phone alarm.<br><br>**Condom use:** She reported consistent condom use throughout the study survey. In the interview she reported two instances of condom rupture, early in the study, but she did not report them in the survey.

	**Number of clients:** The trend from the results of the daily phone study survey appears stable. She reported not sharing information about PrEP use with clients because she anticipated request for condomless sex.
**ID14[c]**	**PrEP adherence:** ID had a response rate of 86.3%. She took PrEP almost all the days in which she replied to the daily survey (97.3%). She only skipped PrEP at the very end because she stopped being a sex worker. She did not need alarms to remember to take PrEP.<br><br>**Condom use:** She reported consistent condom use through the study survey, in the interview she reported continuing using condoms as usual as she was not sure about PrEP efficacy.<br><br>**Number of clients:** The trend from the results of the daily phone study survey appears to sho a decrease in the number of clients. She did not see a relationship between PrEP use and number of clients.
**ID15[c]**	ID had a response rate of 3%.

All percentages in this table refer to the total number of days with response to daily surveys. t= transgender woman, c= cisgender woman

* This percentage refers to the total number of sexual encounters, not to confuse with the percentage of total number of days with condomless intercourse reported through the daily phone study survey.
