# Supplementary material for: Intensive longitudinal follow-up of cisgender and transgender women engaged in sex work during the three months following initiation of daily oral PrEP: A series of case-studies with mixed-method assessments
Source: PLOS Glob Public Health. 2026 May 7;6(5):e0006056. doi: 10.1371/journal.pgph.0006056 (PMC13152121; doi:10.1371/journal.pgph.0006056)
Supplement: Attachment — Submitted filename: Response to reviewers.docx [file pgph.0006056.s006.docx]

**RESPONSE TO REVIEWERS**

**Journal Requirements**

**1. Please amend your detailed Financial Disclosure statement. This is published with the article. It must therefore be completed in full sentences and contain the exact wording you wish to be published.**

**i. State the initials, alongside each funding source, of each author to receive each grant. For example: "This work was supported by the National Institutes of Health (####### to AM; ###### to CJ) and the National Science Foundation (###### to AM)."**

The new funding paragraph reads as follows:

“This work was supported by ‘la Caixa’ Foundation (ID 100010434, fellowship code LCF/BQ/DI20/11780017, awarded to LJVG), the European Union (“NextGenerationEU"/“PRTR”, awarded to GC), the Spanish government (grant RYC2021-033537-I, supported by MCIN/AEI/10.13039/501100011033, awarded to GC), and the Ministry of Research and Universities of the Government of Catalonia (2021 SGR 01563, awarded to GC). This work was also supported by the Spanish government (grant CEX2023-0001290-S, supported by MCIN/AEI/ 10.13039/501100011033, awarded to ISGlobal), and the Generalitat de Catalunya (through the CERCA Program, awarded to ISGlobal). Funders had no role in the study design, data collection, analysis, or interpretation of the results.”

**2. Please ensure that your Ethics Statement is available in its entirety at the beginning of your Methods section, under a subheading 'Ethics Statement'. It must include: 1) The name(s) of the Institutional Review Board(s) or Ethics Committee(s), 2) The approval number(s), or a statement that approval was granted by the named board(s), 3) (for human participants/donors) - A statement that formal consent was obtained (must state whether verbal/written) OR the reason consent was not obtained (e.g. anonymity).**

We have moved the Ethics Statement to the beginning of the Materials and Methods section, under a new subheading *Ethics Statement*. It now includes a sentence confirming that formal written consent was obtained from all participants.

Original statement (Materials and Methods, Study Design and Data collection, line 140-141): “The protocol was reviewed and approved by the Ethics and Research Commission from the Hospital Universitario Infanta Leonor and the Hospital Virgen de la Torre (086-22).”

Updated statement (Materials and Methods section, under a new subheading 'Ethics Statement', lines 124-126): “The protocol was reviewed and approved by the Ethics and Research Commission from the Hospital Universitario Infanta Leonor and the Hospital Virgen de la Torre (086-22) in June 2022. Formal written consent was obtained from all participants.”

**If the reviewer comments include a recommendation to cite specific previously published works, please review and evaluate these publications to determine whether they are relevant and should be cited. There is no requirement to cite these works unless the editor has indicated otherwise.**

**Additional Editor Comments (if provided):**

**The authors are invited to revise the manuscript, and in particular, provide more details surrounding the methodology.**

We thank the Editor for this comment. In the revised manuscript, we have expanded the methodology section to provide additional detail on study eligibility criteria, recruitment procedures, the PrEP clinic appointment process, and the qualitative analysis. These clarifications were incorporated in response to specific reviewer queries.

[Note: HTML markup is below. Please do not edit.]

**Reviewers' comments**

**Reviewer's Responses to Questions**

**Reviewer #1: Thank you for the opportunity to review the manuscript. This manuscript aims to understand the daily experiences of women sex workers using PrEP, with a focus on PrEP adherence. This is an important issue. Given substantial evidence on the effectiveness of PrEP, adherence is currently one of the main focuses of PrEP implementation. I commend the authors for their integrated mixed methods approach to tackle the research question. Below, I raise several points for consideration and/or clarification. In particular, the manuscript could benefit from more details on study methodology.**

**Major revisions**
**Introduction:**

**- Given that the focus of the paper is on women sex workers and the authors stratify results by transgender vs cisgender women sex workers, it would be helpful if the authors could provide more context on: 1) HIV prevalence/incidence among women sex workers rather than in the Spanish population overall [lines 57-58]; and 2) differences in social and structural contexts (e.g., stigma and discrimination, legal/structural barriers) and health risks (e.g., HIV prevalence/incidence, access to care, violence exposure, etc.) between transgender and cisgender women sex workers.**

We agree that this is valuable information to add to the manuscript. With regard to the first point, the following sentence has been added to the Introduction:

New sentence (Introduction, first paragraph, lines 69-72): “HIV prevalence among sex workers is estimated to be 0.8% to 2% among cisgender women (individuals assigned female at birth who identify as women),(6) higher among intravenous drug users and Spanish nationals, and 25% to 27% among transgender women.(8–11)”

With regard to the second point, this is a challenging question to address. Data on sex work are fragmented, often derived from small cohorts and, in some cases, more than a decade old. The absence of disaggregated data by gender identity and sex work status, as well as the lack of direct comparisons between transgender and cisgender women sex workers, further limits the ability to provide a definitive answer. After a careful review and interpretation of the available evidence, some differences emerge in HIV prevalence, condom use, sexualized drug use, and engagement in work‑related travel. In contrast, both groups appear relatively similar in terms of country of origin, workplace settings, experiences of stigma, and prevalence of mental health issues. Importantly, transgender women face additional barriers related to transphobia and limited access to gender‑affirming care. This information has been incorporated into the manuscript as follows:

New paragraph (Material and Methods, Study population, context and inclusion criteria, lines 128-144): “Most available data about sex work in Spain come from women accessing outreach services, which are predominantly independent workers, migrants from Latin America (about half with regularized status), and 35-7% street based.(7,9,11,37) Spanish born women, who represent 5-10% in more recent cohorts,(7,8,11) are disproportionately represented among those affected by substance dependence and homelessness, particularly among cisgender sex workers.(13,37) Condom use with clients among cohorts ranges from 75% to 100%.(11,13,14,38–40) Inconsistent use among cisgender women is linked to being Spanish born, substance dependence, violence, prior condom rupture, and a history of sexually transmitted infections (STI);(11,38,39,41) while among transgender women it is more often reported in private sporadic encounters.(9) In both groups, condom rupture and coercion with clients are common even among those reporting consistent use.(9,38) Cocaine is widely consumed in professional contexts due to client demand, while transgender women additionally report use of Viagra, poppers, and less frequently substances such as GHB, MDMA, or methamphetamine.(9) Transgender women are also reported to engage in national and international work related travel.(9) Stigma, violence, and mental health issues are widespread in both communities, with transgender women facing added barriers due to transphobia.(9,42)”

**- Lines 60-61: The authors state that PrEP is subsidized. It would be useful for the authors to provide more details on subsidized access to PrEP. E.g., What proportion of PrEP costs is subsidized or are all PrEP costs covered? This information can help contextualize the role of cost on PrEP access/adherence.**

We appreciate this comment. We have clarified that PrEP is a fully subsidized program delivered through the national health system for individuals meeting the Ministry of Health’s eligibility criteria.

Information about the subsidized nature of the program has been added to the Introduction:

Original sentence (Introduction, line 59-61): “…Spain recognized sex workers as a population at high risk of acquiring HIV and supported subsidized access to pre-exposure prophylaxis (PrEP) in 2019.[3]”

Updated sentence (Introduction, lines 60-62): “To curb the epidemic, in 2019 Spain introduced fully subsidized pre-exposure prophylaxis (PrEP) for individuals at high risk, including sex workers.”

Information about criteria for PrEP access has been added to the Material and methods:

New paragraph (Material and Methods, Study population, context and inclusion criteria, lines 145-151):” Within the national healthcare system, women sex workers are eligible for PrEP if they disclose their sex work status and report inconsistent condom use.(12) Both transgender and cisgender women sex workers may also qualify without disclosure if they report at least two high risk behaviors in the past year, with cisgender women additionally required to report inconsistent condom use. High risk behaviors include having more than ten sexual partners, condomless anal sex, drug use associated with unprotected sex, repeated use of post exposure prophylaxis, or a diagnosis of a bacterial STI.(12)”

**Methods:**

**- More information on study setting/venue (e.g., was venue located in an urban, suburban, or rural region?) and how participants were recruited to the study (e.g., active recruitment via staff at the PrEP clinic or passive recruitment via posters/ads at the PrEP clinic) are needed. If available, it would also be interesting to see a breakdown of how many cisgender or transgender women presented at the PrEP clinic, how many were sex workers, how many were eligible for study participation, and, of these, how many accepted or declined to participate in the study. These numbers may speak to potential selection bias in the study and the representativeness of the study sample.**

We appreciate this question and agree that this information strengthens the assessment of the study, its applicability to other contexts, and potential bias. We have added details about the clinic and recruitment process.

Original statement (Material and Methods, Study design and data collection, lines 122-123): “Participants were screened and enrolled consecutively in the order they presented to the PrEP clinic during a fixed recruitment period from November 8^th^, 2022 to January 30^th^, 2023”

Updated statement (Material and Methods, Study design and data collection, lines 162-168): “Participants were screened and consecutively enrolled in the order they presented to a suburban hospital-based PrEP clinic during a fixed recruitment period from November 8, 2022 to January 30, 2023. The NGOs Madrid Positivo and In Género, collaborators in prior projects, informed women sex workers in their networks about the clinic and provided practical guidance on how to attend. Some participants were already familiar with PrEP and the clinic’s services through earlier fieldwork surveys assessing interest in PrEP.(13,14) Peer referral later expanded participation beyond the initial NGO network.”

Even though we do not have information on the total number of cisgender or transgender women presenting to the clinic, but we do have data on the number of sex workers that attended the clinic between October 2022 and August 2023. During this period, 60 sex workers initiated PrEP at the study clinic (9 cisgender women, 33 transgender women, and 18 cisgender men) (Valencia et al., 2024). We have used this information on clinic attendance after study initiation to strengthen the argument that our sample is representative of clinic patterns:

Original paragraph (Discussion, Strength, limitations and perspective, lines 637-640): “Firstly, the small number of participants prevents us from generalizing the results to other populations and to perform a formal comparison between cisgender and transgender women sex workers. Nevertheless, participants were enrolled in the order in which they presented to the PrEP clinic where the study was carried at. Therefore, this is a small but probably representative sample of the sex workers attending the clinic where the study took place.

Updated paragraph (Discussion, Strength, limitations and perspective, lines 561-565): “Enrollment figures were also consistent with clinic attendance patterns between 2022 and 2023 (approximately 4.2 women sex workers per month, cisgender to transgender ratio 0.2:1),(63) suggesting representativeness.”

Additionally, the manuscript contains information about the number of women sex workers approached who accepted to participate in the study**:**

Original sentence (Results, demographics, lines 274-276): A total of 16 women sex workers were approached for recruitment for the study. Finally, 15 participants (12 transgender and 3 cisgender women) aged between 23-43 years old were enrolled between October 2022 and January 2023

**- Lines 114-116: It is unclear how the research team identified cisgender or TGW and sex workers? For instance, did participants need to self-report as a cisgender women or TGW sex worker or was there an existing database to draw from? It was also unclear how potential participants’ ability “to attend medical visits” was assessed at baseline? It would be helpful if these details could be included in the manuscript.**

The first part of this question is addressed in our response above. As explained, women sex workers learned about the PrEP clinic through collaborating NGOs, earlier fieldwork surveys conducted by the study team, or peers already attending the clinic. Upon presenting to the clinic, they informed staff why they were there and how they had learned about the clinic, although this information was anecdotal. In addition, all individuals presenting to the PrEP clinic were routinely asked about risk factors qualifying them for fully subsidized PrEP under the national healthcare system. These data, including engagement in sex work, are recorded in the national PrEP surveillance system (SiPrEP). For study enrollment, participants were required to self‑identify as sex workers during their initial PrEP visit. No external database was used to identify or classify participants.

Regarding the assessment of participants’ ability to attend medical visits, during the initial PrEP encounter all individuals interested in starting PrEP were informed of the mandatory schedule of clinical visits required for initiation and continuation. At that time, they were asked to verbally confirm that they had the time and means to comply with these visits. It is important to note that medical visits, laboratory testing, and pharmacy dispensation could not always be completed on the same day, which made this confirmation particularly relevant.

This information was added to the manuscript as follows:

Original statement (Material and Methods, Study design and data collection, lines 122-123): “Participants were screened and enrolled consecutively in the order they presented to the PrEP clinic during a fixed recruitment period from November 8^th^, 2022 to January 30^th^, 2023”

Updated statement (Material and Methods, Study design and data collection, lines 162-168): “Participants were screened and consecutively enrolled in the order they presented to a suburban hospital-based PrEP clinic during a fixed recruitment period from November 8, 2022 to January 30, 2023. The NGOs Madrid Positivo and In Género, collaborators in prior projects, informed women sex workers in their networks about the clinic and provided practical guidance on how to attend. Some participants were already familiar with PrEP and the clinic’s services through earlier fieldwork surveys assessing interest in PrEP.(13,14) Peer referral later expanded participation beyond the initial NGO network.”

New paragraph (Material and Methods, Study design and data collection, lines 174-178): “They also receive information about the schedule of follow‑up appointments, which encompass medical evaluations, laboratory testing, and PrEP dispensation at the hospital pharmacy. In the context of this study, all women enrolled self‑identified as sex workers at the initial visit and confirmed that they had the means and time to attend the scheduled appointments.”

**- It would be valuable for the authors to include a reflexivity of the research team detailing how interviewers' and analysts' backgrounds may have impacted data collected and the themes identified.**

We appreciate the reviewer’s suggestion and have now added a reflexivity statement to clarify how the research team’s backgrounds may have influenced data collection and analysis. This information has been included under limitations:

New paragraph (Discussion, Strength, limitations and perspective, lines 598-609): “Finally, the qualitative results may have been influenced by researchers’ backgrounds. Interviews were conducted by a medical student, a pharmacist, and an infectious diseases physician who trained in qualitative interview techniques. The physician was also responsible for enrolling participants, which may have affected how some women responded; however, her clinical experience and long‑standing work with this population enabled more nuanced, context‑sensitive questioning. The decision to include the physician as an interviewer was made to address scheduling difficulties between some participants and the other two interviewers, as well as to provide an opportunity for her to learn qualitative methods as part of her PhD program. Qualitative analysis was carried out jointly by a specialist in qualitative research methods and the same infectious diseases physician, combining methodological rigor with in‑depth familiarity with PrEP, HIV prevention, and the lived realities of women sex workers. The full research team reviewed and discussed emerging findings throughout the analytic process.”

**- It is unclear if the sample size was predetermined or if the authors kept recruiting until they reached saturation in the qualitative interviews? It would be helpful if these details could be included in the manuscript.**

We appreciate this comment. Participants were screened and consecutively enrolled in the order they presented to a suburban hospital‑based PrEP clinic during a fixed recruitment period (from November 8th, 2022 to January 30th, 2023). This is stated in the Methods section, and we have now also included it under the study’s limitations:

New sentence (Discussion, strengths, limitations and perspective, lines 609-611): “Finally, participant enrollment occurred within a fixed recruitment period rather than continuing until thematic saturation was reached, which may have limited the breadth of perspectives captured.”

**Discussion**
**- Lines 640-641: It is difficult to determine if the small sample included in the study was “representative” of the sex workers attending the clinic where the study took place as it is unclear how the research team identified sex workers at this clinic. For example, if sex workers were identified via self-report and numerous sex workers chose not to identify themselves as a sex worker, then the study sample would not be representative of sex workers attending the clinic. It may be advisable for the authors to reword their statement.**

We appreciate the opportunity to clarify this point. Because of the referral network established through NGOs and word of mouth among colleagues, we believe that sex workers attending the clinic likely felt comfortable disclosing their sex work status. However, we cannot guarantee that all sex workers revealed their status, as subsidized PrEP services could also be accessed through a combination of other reported risk factors (as described in the revised manuscript under *Study population, context, and study criteria,* lines 145 to 151). This combination of organizational familiarity and community‑level awareness was critical; without it, it is unlikely that women sex workers would have presented to the clinic at all, based on data from other national PrEP clinics.

Prior data from two large STI clinics in Barcelona (Ugarte et al., 2023) and Madrid (Piñeiro et al., 2025) reported very low numbers of transgender women, cisgender women, and sex workers accessing services (18 TGW and 4 cisgender women in a two‑year cohort of 2,256 PrEP users in Madrid, and 14 TGW, 5 cisgender women, and 29 sex workers in a two‑year cohort of 297 PrEP users in Barcelona). In contrast, during the 10 months following the onset of this study, a total of 33 transgender women sex workers, 9 cisgender women sex workers, and 18 cisgender male sex workers initiated PrEP at the study clinic (Valencia et al., 2024). The cisgender‑to‑transgender sex worker ratio (0.2:1) closely mirrors the ratio observed among study participants, suggesting that our sample is representative of those presenting to this clinic, though not necessarily of the broader population.

Information about how sex workers were identified for enrollment has been included in the methodology:

New sentence (Material and methods, Study design and data collection, lines 162-168): “Participants were screened and consecutively enrolled in the order they presented to a suburban hospital-based PrEP clinic during a fixed recruitment period from November 8, 2022 to January 30, 2023. The NGOs Madrid Positivo and In Género, collaborators in prior projects, informed women sex workers in their networks about the clinic and provided practical guidance on how to attend. Some participants were already familiar with PrEP and the clinic’s services through earlier fieldwork surveys assessing interest in PrEP.(13,14) Peer referral later expanded participation beyond the initial NGO network.”

New paragraph (Material and Methods, Study design and data collection, lines 169-178): “The initial PrEP clinic visit routinely includes assessment of risk factors for HIV infection that qualify individuals for fully subsidized PrEP under the national health system, such as engagement in sex work; this information is subsequently submitted to the national surveillance program for PrEP use.(12)… …In the context of this study, all women enrolled self‑identified as sex workers at the initial visit and confirmed that they had the means and time to attend the scheduled appointments.”

Information that supports the robustness of the findings and why we believe the sample to be representative of the population presenting to the clinic has been added to the manuscript Limitations section:

Original paragraph (Discussion, Strength, limitations and perspectives, lines 637-640): “Firstly, the small number of participants prevents us from generalizing the results to other populations and to perform a formal comparison between cisgender and transgender women sex workers. Nevertheless, participants were enrolled in the order in which they presented to the PrEP clinic where the study was carried at. Therefore, this is a small but probably representative sample of the sex workers attending the clinic where the study took place.

Updated paragraph (Discussion, Strength, limitations and perspective, lines 557-567): “Firstly, the small number of participants limits the generalizability of our findings and precludes formal comparison between cisgender and transgender women sex workers. Nevertheless, each participant contributed a substantial amount of data (a total of 1,266 daily survey responses were collected), which strengthens the robustness of the findings. Enrollment figures were also consistent with clinic attendance patterns between 2022 and 2023 (approximately 4.2 women sex workers per month, cisgender to transgender ratio 0.2:1),(63) suggesting representativeness. Moreover, attendance at this clinic was also higher than in other national cohorts,(17,64) likely reflecting the strength of the referral network. Because of this we hypothesize that sex workers attending the clinic likely felt comfortable disclosing their status, although we cannot assume all did so, as subsidized PrEP could also be accessed through other risk factors”

**- The authors noted in the Results section that all participants were from Latin America. It may be helpful to add a few lines in the Discussion to consider the potential implications of this. E.g., how the cultural backgrounds of participants may influence their perceptions of PrEP, how they acquire PrEP knowledge, adherence, etc. It may also be helpful for authors to contextualize their findings in a landscape of expanding PrEP modalities where users may not need to take PrEP daily (e.g., event-driven PrEP, long-acting injectable PrEP).**

We appreciate this suggestion. It is difficult to report precisely how cultural background may have influenced participants, since PrEP perceptions among cisgender and transgender women sex workers in Latin America remain under‑researched. Cultural influences also differ across countries, reflecting both social contexts and the varying stages of national PrEP program development. Women in our study originated from Cuba, Ecuador, Colombia, Costa Rica, El Salvador, and Brazil, where PrEP implementation is uneven: Brazil and Cuba have established national programs offering free oral PrEP, while Colombia and Ecuador have approved PrEP but with modest coverage, largely concentrated in NGO‑supported projects. Costa Rica only approved PrEP in 2021, and El Salvador has recently published technical guidelines (2024) but is still in early rollout.

Migrants, particularly those with irregular status and coming from contexts with limited social and structural support, may be more likely to seek information through informal channels due to lack of knowledge of official services or mistrust of institutions. Experiences of stigma, violence, and hate crimes in their countries of origin may further heighten their perception of vulnerability and shape how they engage with prevention services in Spain.

It is possible that if Spanish‑born women had been enrolled in the study, they might have acquired PrEP knowledge through different channels and displayed distinct adherence patterns. Notably, Spanish‑born women are over‑represented among individuals experiencing unstable housing and drug dependence, making them particularly difficult to engage through traditional healthcare systems

This information has been included in the manuscript as follows:

New paragraph (Discussion, Strength, limitations and perspective, lines 582-589): “Thirdly, the study sample consisted exclusively of women from Latin America, an under‑researched group in terms of PrEP perceptions. Migrant women, particularly those with irregular status, may rely on informal information channels due to mistrust of institutions or limited knowledge of official channels.(66,67) Experiences of stigma and violence in their home contexts may further heighten vulnerability and shape engagement with prevention services in Spain.(68) Spanish‑born women, who represent only a minority of sex workers reached through NGOs,(69) may have acquired PrEP knowledge through different channels and displayed distinct adherence patterns.”

With regards to the second point, we recognize that our findings should be interpreted within the broader context of expanding PrEP modalities. In this manuscript, we focus on daily oral PrEP, as it is currently the only modality approved by the European Medicines Agency (EMA). Event‑driven PrEP has not been approved by the EMA, nor by any international guideline for cisgender or transgender women, due to insufficient evidence, although future studies may help clarify its role. Injectable PrEP, approved by the EMA in 2025 for all populations at risk of HIV, is not yet widely implemented in Spain. Preferences for PrEP delivery modalities among cisgender and transgender women sex workers were described in previous manuscripts by this research team, showing a clear preference for injectable PrEP (Vazquez et al., 2024; Vazquez et al., 2025), which (if available) would likely facilitate adherence and offer greater discretion, as it does not depend on daily routines or the presence of pills that may be visible to others at home or work. However, even with long‑acting injectables, adherence can be challenging: the ENCORE study among transgender women in the United States and Puerto Rico reported that 17% were at least one week late and 13% at least four weeks late to their next injection (Conney et al., 2024). Offering oral pills has therefore been highlighted as a useful strategy to bridge gaps in coverage between injections (Landovitz et al., 2023).

To date, only two studies have examined longitudinal PrEP trajectories among cisgender and transgender women sex workers. The first, in South Africa, reported low persistence with frequent cycling on and off PrEP, but did not clarify whether interruptions reflected changes in HIV risk (Rao et al., 2023). The second, in the United States, identified four typologies of discontinuation among transgender women, including PrEP cycling, which generally aligned with shifts in HIV acquisition risk linked to socio‑behavioral changes such as disengagement from and re‑engagement in sex work. Importantly, this trajectory was not associated with HIV seroconversion, suggesting that it may represent a prevention‑effective adherence pattern (Cooney et al., 2023).

Our current study adds insight into longitudinal patterns of use of daily oral PrEP. Only one participant discontinued PrEP entirely, coinciding with her exit from sex work. Some participants discontinued PrEP for short periods in the setting of illness, medical procedures, or breaks in sex work. Participants tended to abstain from sex during extended gaps in PrEP use, exercising caution upon resuming the medication. Our findings highlight that women sex workers are capable of making informed decisions about PrEP use, modulating adherence according to perceived HIV risk, cycling off during lower‑risk periods, and respecting lead‑in times before resuming high‑risk activity. These results should be interpreted within the supportive context of an observational study with adherence monitoring. Continued collection of this type of information will remain important even as injectable PrEP becomes more widely implemented, since adherence gaps between injections are possible (Conney et al., 2024). Such data can inform appropriate counseling and support, and may also contribute to evaluating the potential role of event‑driven PrEP if regulatory agencies approve its use among cisgender and transgender women.

We have added information about the study among transgender women in the United States providing longitudinal PrEP trajectories as follows:

Original paragraph (Discussion, PrEP adherence and side effects, lines 548-551): “Current literature shows that PrEP adherence among women sex workers is usually low, with high rates of early discontinuation. One longitudinal study among female sex workers in South Africa also described frequent cycling on and off PrEP amongst their participants.[17,36,37]”

New paragraph (Discussion, PrEP adherence and side effects, lines 446-456): “Current literature shows that PrEP adherence among women sex workers is usually low, with high rates of early discontinuation.(17,36,37) To date, only two studies have examined longitudinal PrEP trajectories among cisgender and transgender women sex workers. One longitudinal study among female sex workers in South Africa described frequent cycling on and off PrEP amongst their participants, although it did not clarify whether interruptions reflected changes in HIV risk.(51) The second, in the United States, described several distinct patterns in how transgender women stopped and restarted PrEP, One of these patterns was ‘PrEP cycling’, which tended to follow changes in HIV risk linked to shifts in socio behavioral circumstances, such as moving out of and back into sex work. Importantly, this trajectory was not associated with HIV seroconversion, suggesting that it may represent a prevention-effective adherence pattern.(52)”

We have also detailed how our manuscript expands the evidence base on PrEP trajectories among women sex workers, and why sustained collection of similar data will be important for evaluating new PrEP modalities as they are introduced:

Original paragraph (Discussion, PrEP adherence and side effects, lines 551-558): “To our knowledge, this is the first study providing insight into day-to-day adherence patterns among women sex workers. Despite variable levels of adherence, participants’ intake was sufficient to preserve efficacy (4 or more doses per week).[10,13] This is true because missed doses were randomly distributed during the study period, and when participants missed PrEP for several consecutive days they did not engage in sex work and remained cautious after restarting the medication. Nevertheless, we identified some opportunities to boost adherence among study participants.”

Updated paragraph (Discussion, PrEP adherence and side effects, lines 459-474): “Our current study adds insight into longitudinal patterns of daily oral PrEP use among women sex workers. Only one participant discontinued PrEP entirely, coinciding with her exit from sex work. Despite variable levels of adherence, participants’ reported PrEP intake appeared sufficient to preserve efficacy (four or more doses per week).(10,13) Participants appeared to practice prevention-effective adherence: missed doses were randomly distributed, and when participants missed PrEP for several consecutive days, they did not engage in sex work and remained cautious after restarting the medication. These findings highlight that women sex workers are capable of making informed decisions about PrEP use, modulating adherence according to perceived HIV risk, cycling off during lower risk periods, and respecting lead-in times before resuming high risk activity. These findings also suggest that early adherence challenges are driven by day‑to‑day circumstances rather than progressive disengagement. These results should be interpreted within the supportive context of an observational study with adherence monitoring. Continued collection of this type of information will remain important even as injectable PrEP products are implemented, since adherence gaps between injections will be threats to efficiency.(52) Such data can inform appropriate counseling and support, and may also contribute to evaluating the potential role of event driven PrEP use among cisgender and transgender women (not yet approved by regulatory agencies).”

Finally, we have mentioned that injectable PrEP, which was recently approved by the EMA, is likely to improve adherence among the study population:

New sentence (Discussion, lines 490-493): “The future integration of long‑acting formulations into PrEP clinics, recently approved by the European Medicines Agency in 2025, is a promising strategy to reduce nonadherence related to forgetfulness and routine disruptions.(54)”

**Minor revisions**
**Abstract:
- Given the number of existing and emerging PrEP modalities, it would be helpful to specify/include “daily oral PrEP” in the title, abstract, and objective statement.**

We appreciate and agree with this suggestion, the following changes have been included to the title and the abstract:

Original title: “Intensive longitudinal follow-up of cisgender and transgender women engaged in sex work during the three months following PrEP initiation: a series of case-studies with mixed-method assessments”

Updated title: “Intensive longitudinal follow-up of cisgender and transgender women engaged in sex work during the three months following initiation of daily oral PrEP: a series of case-studies with mixed-method assessments”

Original sentence (Abstract, line 36): “…daily assessments of self-reported use PrEP use,”

Updated sentence (Abstract, line 36): “…daily assessments of self-reported use of daily oral PrEP,”

Original sentence (Abstract, line 55-56): “We found a complex interplay of individual, occupational, and structural factors shaping early PrEP adherence among participants.”

Updated sentence (Abstract, line 55-56): “We found a complex interplay of individual, occupational, and structural factors shaping early adherence to daily oral PrEP among participants.”

**- It is unclear what the authors mean by “dynamics of PrEP adherence” in the abstract (lines 34-35) and introduction (line 108). Given this is a key outcome of the study, it would be helpful if the authors could define the term in the abstract and introduction.**

We agree with this recommendation. The use of the term has been modified in the abstract as follows:

Original sentence (Abstract, line 34-35): “…with emphasis on understanding the dynamics of PrEP adherence.”

Updated sentence (page 2, paragraph 1, line 34-35): “…with emphasis on understanding the dynamic process of initiating and sustaining PrEP adherence”

The term “dynamics” has been removed from the introduction to allow for a more detailed explanation and to avoid ambiguity. In addition, we have introduced an explanation of the Medication Adherence Model (MAM), as recommended by another reviewer.

Original paragraph (Introduction, lines 100-109): “Little is known about the dynamics of PrEP adherence and the potential behavioral changes associated with its initiation in women sex workers. To better understand this, we conducted an intensive longitudinal study in which self-reported PrEP adherence, side effects, condom use, and number of clients were monitored daily across three months via a smartphone app among women sex workers in Madrid, Spain, after initiating PrEP. This was followed by an in-depth explanatory qualitative interview that also explored initiation, PrEP communication with clients and coworkers, and PrEP stigma. This study aims to provide an in-depth, daily account of the lived experiences of woman sex workers using PrEP, with special emphasis on understanding the dynamics of PrEP adherence in order to inform future interventions.”

Updated paragraph (Introduction, lines 103-121): “Originally developed to guide clinicians in evaluating adherence among patients with hypertension, the Medication Adherence Model (MAM) provides a useful framework for understanding how individuals make and sustain medication taking decisions.(36) Through its three core concepts (Purposeful Action, Patterned Behavior and Feedback), the model provides a structured lens for examining what motivates individuals to take or skip medication, how daily patterns form over time, and how these patterns interact with broader structural and occupational contexts such as sex work. Moreover, MAM’s distinction between intentional nonadherence (e.g., cycling off during perceived low risk periods) and unintentional nonadherence (e.g., forgetting doses or facing access barriers) is particularly relevant for providers, as it highlights different points of intervention to support adherence. Guided by this framework, we conducted an intensive longitudinal study to better understand the phenomenon of PrEP adherence and its bidirectional relationship with sex work. Self-reported PrEP adherence, side effects, condom use, and number of clients were monitored daily for three months via a smartphone app among women sex workers in Madrid, Spain, after initiating PrEP. This was followed by an in-depth explanatory qualitative interview that also explored initiation, PrEP communication with clients and coworkers, and PrEP-related stigma. This study aims to provide an in-depth, daily account of the lived experiences of women sex workers using PrEP, generating insights that can inform future interventions.”

**- Line 38: The term “communication” is a bit vague. Specifying “communication between sex workers and their clients” would help improve clarity.**

We agree with this suggestion. The following changes have been introduced in the abstract:

Original sentence (Abstract lines 38-39): “…qualitative interviews that also explored PrEP initiation, communication, and stigma”

Updated sentence (Abstract, line38-39): “…qualitative interviews that also explored PrEP initiation, communication with sex workers and clients, and stigma”

In addition, we reviewed the remainder of the manuscript and confirmed that in all other instances where communication is mentioned, it is explicitly specified as occurring with sex workers and clients; therefore, no further changes were required.

**Introduction:
- Line 57-58: If data are available, stating how HIV incidence in Madrid compares to Spain and/or the rest of Europe would be valuable.**

We agree with this recommendation. The following changes have been introduced in the Introduction of the manuscript:

New sentence (Introduction, lines 59-60): “with the Community of Madrid consistently recording the highest number of new HIV diagnoses nationwide.(3)”

**- Lines 76-78 state that, “adherence to PrEP with four or more doses per week is likely to provide optimal HIV protection for most individuals,[9] including cisgender women.[10]” Is this statement also true for transgender women?**

Yes, including transgender women. This sentence was intended to emphasize novel findings showing that, after years of rigid recommendations for perfect adherence among cisgender women, emerging evidence now suggests that non‑daily adherence may still confer meaningful protection. To avoid ambiguity, we have updated the sentence to remove the specification of cisgender women.

Original sentence (Introduction, lines 76-78): “After initiation, research shows that adherence to PrEP with four or more doses per week is likely to provide optimal HIV protection for most individuals,[9] including cisgender women.[10]”

Updated sentence (Introduction, page, paragraph, lines 78-80): “Once initiated, adherence of four or more doses per week is considered sufficient to provide optimal HIV protection among cisgender and transgender women.(15,16)”

**- Lines 87-88: It would be helpful if these findings were among the sex work population or other populations.**

The reviewer is referring to the sentence: “Importantly, PrEP use has been associated with a decrease in condom use and/or an increase in the number of sexual partners.(25,26)” This statement draws on evidence from men who have sex with men. However, such findings must be interpreted with caution, as enhanced surveillance and improved STI control should be considered when analyzing STI trends in the PrEP era.

Later in the paragraph, we explain that evidence of behavioral changes after PrEP initiation among women sex workers is limited. We originally cited one study from Benin among cisgender women sex workers. Since writing the manuscript, we have identified an additional study by Phanuphak et al., (2018) among transgender women sex workers in Thailand, which found no increase in reported number of sexual partners, condomless sex, or drug use over 12 months of PrEP use, and importantly, it documented a decrease in syphilis prevalence at six and 12 months compared to baseline.

To incorporate this new evidence, we have modified the manuscript as follows:

Original sentence (Introduction, lines 90–93): “The evidence for risk‑compensating behaviors among cisgender and TGW sex workers is scant, with just one prospective PrEP observational study in this topic carried out in Benin that failed to show evidence of risk compensation among female sex workers after two years of follow‑up.[17]”

Updated sentence (Introduction, lines 91-94): “To date, two prospective studies conducted in Benin and Thailand among cisgender and transgender women sex workers, respectively, have found no evidence of reduced condom use following PrEP initiation and have documented declines in STI diagnoses over the follow‑up period.(33,34)”

Because of the addition of this new reference, we also modified a sentence in the discussion:

Original sentence (Discussion, lines 595-597): “The report of the cisgender women in our study is in concordance with the lack of risk compensating behaviors identified in the only study on this area carried amongst female sex workers in Benin.[41]”

New sentence (Discussion, lines 514-151): “The report of the three cisgender women in our study aligned with prior studies that show no evidence of decreased condom use after PrEP initiation among women sex workers.(33,34)”

**- Line 96: It is unclear which behaviors the authors are referring to here as many have been mentioned so far.**

The reviewer is referring to the following sentence (Introduction, lines 96-99): “Learning about these behaviors is important to understand how they shape PrEP adherence…”. We have modified this sentence as follows

Updated sentence (Introduction, lines 98-102): “Learning about changes in condom use, the number of clients, and communication patterns is important to understand their relationship with PrEP adherence and to inform multifaceted HIV prevention programs that address PrEP-associated behavioral spillovers through appropriate messaging, STI care, family planning services, and empowerment.”

**Methods:
- There are inconsistency in recruitment dates: October 2022 to January 2023 (line 40) in Abstract vs November 8, 2022 and January 2023 in Methods (line 123-124) and October 2022 and January 2023 in Results (line 211). Please ensure recruitment dates are accurate and consistent across sections.**

We appreciate this correction. The correct recruitment dates are November 8, 2022 and January 2023. The information has been updated in the Abstract:

Original sentence (Abstract, line 40): “…between October 2022 and January 2023,…”

Updated sentence (Abstract, line 41): “…between November and January 2023,…”

And the Results section:

Original sentence (Results, line 211): “…between October 2022 and January 2023.”

Updated sentence (Results, line 276): “…between November 2022 and January 2023.”

**- Line 196: It would be helpful for authors to specify what they did for content analysis.**

We appreciate this comment. We have expanded the description of our qualitative analysis in the following paragraph and corrected the terminology to specify that we conducted a hybrid deductive-inductive thematic analysis, which is a thematic analysis incorporating elements of content analysis.

Original paragraph (Material and methods, Data analysis, Qualitative analysis, lines 194-202): Interviews were transcribed and coded in Spanish and later translated into English to facilitate review and feedback by non-Spanish speaking members of the research team. Content analysis of transcripts was used to identify participant’s experiences after starting PrEP. LVG developed the original codebook after a careful reading of the transcripts. CV and LVG applied the codebook to the transcripts. The resulting kappa coefficient for each interview was ≥ 0.72.[30] Data on the topics addressed in the current study was comprehensively summarized by LVG and CV. At each step of the process, disagreements were discussed until a consensus was reached. MAXQDA 24[31] was used for coding.

New paragraph (Material and methods, Data analysis, Qualitative analysis, lines 259-267): A hybrid deductive-inductive thematic analysis was performed. LVG created the coding framework in two phases: (1) main categories were established deductively from literature review and research aims, and (2) subcategories emerged inductively through preliminary analysis of interview transcripts. LVG and CV subsequently conducted the comprehensive qualitative analysis in MAXQDA,[31] systematically applying the codebook, identifying patterns and relationships between codes, and interpreting themes across all interviews. The resulting kappa coefficient for each interview was ≥ 0.72.[30] Data on the topics addressed in the current study was comprehensively summarized by LVG and CV. At each step of the process, disagreements were discussed until a consensus was reached.

.
**- Line 128: GDPR acronym was not defined.**

We appreciate this correction, the definition has been incorporated as follows:

Original sentence (Methods, Study design and data collection, lines 128-129)**:** “…GDPR-compliant application *m-path* application…”

Updated sentence (Methods, Study design and data collection, lines 184-185): “…M-path is a GDPR (General Data Protection Regulation) compliant application…”

**Results:
- Line 339: The authors state that “When participants missed PrEP for one to three days in a row, it was usually due to side effects and/or work related issues.” It would be helpful to provide a statistic instead of using the word “usually”.**

We cannot provide an exact statistic as requested by the reviewer. Exit interviews did not offer day‑by‑day accounts that could be directly matched to each daily survey entry and forcing a direct alignment between the two data sources would therefore risk over‑interpretation. In contrast, we can confirm that in the three instances where participants acknowledged missing PrEP for more than three consecutive days, the reasons were illness and a scheduled surgical procedure, not side effects or work‑related issues. These findings are detailed in Table S3 (Joint display table with individual reports of PrEP use and side effects) in the supplementary material. Moreover, we have restructured the Results section according to the suggestions from Reviewer #3, and this information has been rewritten as follows:

New sentences (Results, PrEP adherence and side effects, lines 324-333): “In contrast, participants with irregular adherence described side effects and work-related factors such as long night shifts, substance use, and travel as barriers to maintaining routines, mirroring the scattered missed doses observed in the time‑series data. Intentional non-adherence also emerged, often motivated by concerns about mixing PrEP with alcohol, recreational drugs, or other medications; by wanting to avoid PrEP-related side effects that would otherwise difficult their work (i.e. diarrhea); or by avoiding doses taken “too close together”. Recreational drug use was more commonly associated with intentional nonadherence than with forgetfulness. Longer interruptions (more than 3 days) were related to illness, and for one patient a scheduled surgical procedure.”

**- Lines 348-349 state that, “The average was 32% and 65% for transgender and cisgender women sex workers, respectively (S1 Table).” It is unclear how 65% was calculated if there were only 3 cisgender FSW? It would be helpful if the authors double checked their %’s throughout the manuscript for accuracy.**

We appreciate the reviewer for catching this error. The percentages have been reviewed throughout the manuscript for accuracy and the following corrections have been made, all in the Results section:

Original sentence (Results, PrEP adherence, lines 267-269): “The lowest reported PrEP adherence by a participant during the study period was 37% and the highest 100% (74% and 71% in average for transgender and cisgender women sex workers, respectively).”

Updated sentence (Results, PrEP adherence, lines 307-308): “Individually reported PrEP adherence ranged from 37% to 100% (mean 68% among transgender women and 89% among cisgender women; S1 Table).”

Original sentence (Results, Side effects, lines 346-349): “…the highest reported prevalence of side effects by a participant was 53.3%, meaning she had side effects 24 out of 45 days in which she answered the questionnaire. The average was 32% and 65% for transgender and cisgender women sex workers, respectively (S1 Table).”

Updated sentence (Results, Side effects, lines 310-313): “Side effects prevalence ranged from 0% to 66% (mean 26.4% among transgender women and 32.9% among cisgender women; S1 Table)”

Original sentence (Results, Condom use, lines 405-406): “At the group level, the average was 65% for transgender and 100% for cisgender participants…”

Updated sentence (Results, Condom use, lines 355-357): “Across the study period, condom use varied widely between participants, ranging from 9.3% to 100% (mean 57% among transgender women and 100% among cisgender women; S1 Table)”

**Other notes:
- The manuscript could benefit from a spell check as there are several grammatical errors throughout (e.g., line 79 should state “over time” instead of “overtime”, line 133 should state “were” instead of “where”, line 185 should state “latter” instead of “later”, line 564 should state “coping” instead of “copying”, among others).**

We appreciate this comment and have carefully reviewed the latest version of the manuscript for grammatical accuracy. In addition to correcting the specific errors noted, we conducted a comprehensive grammar and style review across the entire text to ensure consistency, clarity, and fluency.

**Reviewer #2: The manuscript is well-written, easy to follow, and demonstrates a clear understanding of the subject matter. The authors have provided adequate references to support their arguments, which enhances the credibility and scholarly depth of the paper. The structure is logical, and the flow of ideas allows readers to easily grasp the main objectives and findings of the study. However, there is room for improvement, particularly in ensuring that the language used is community-friendly and inclusive. The terminology should reflect the preferences and lived realities of the populations discussed. For instance, instead of using the term “woman sex workers,” the authors are encouraged to adopt “female sex workers,” which is more respectful and widely recognized within community and research contexts. Furthermore, it would be helpful for the authors to define and simplify technical terms such as “cisgender” to make the manuscript more accessible to diverse readers, including those without extensive academic or gender studies backgrounds. In addition, the paper would be strengthened by integrating a more comprehensive review of previous studies, particularly those focusing on similar populations or regional contexts. Highlighting past research will help situate the current study within the broader field, showing its contribution, gaps addressed, and relevance to ongoing discussions in community health and rights. Overall, the manuscript is promising and can make a valuable contribution once these adjustments are made.**

We sincerely thank Reviewer #2 for their thoughtful comments and constructive suggestions. Their feedback has helped us clarify key aspects of the manuscript and strengthen the presentation of our findings. We have carefully considered each point raised and incorporated the recommended changes into the revised version.

With regard to nomenclature, and following the example of the *UnPrEPared* document by the European Sex Worker’s Rights Alliance, we chose the terms *cisgender women sex worker* and *transgender women sex worker* to refer to these groups individually. However, this document does not establish a unified collective term for referring to both groups together and we did not find a precedent in the literature.

Identifying an appropriate term to collectively describe cisgender and transgender women sex workers has therefore been a challenge, discussed extensively within and beyond our research group both before submission and again in response to Reviewer #2’s comment. During data analysis, when presenting findings to colleagues working with LGBTQ+ communities, confusion arose when we used *female sex workers* to refer to both cisgender and transgender women. They encouraged us to adopt an alternative phrasing. Moreover, while it would be ideal to use participants’ own terminology, linguistic differences complicate this process. In Spanish, participants identified as *trabajadora sexual*, where the suffix *‑a* denotes feminine gender/sex, and as *mujer*, a word that refers to both gender and sex. Unlike English, Spanish does not distinguish between *woman* and *female* in everyday usage, and the literal equivalent of *female* (*hembra*) is rarely applied to people. Given these linguistic and conceptual considerations, the closest and most accurate translation of participants’ self‑identification is “women sex workers.”, and if considered appropriate by Reviewer #2, we would prefer to keep this term.

We have added a description of the terms cisgender and transgender in the introduction, after the first use of each term:

Original sentence (Introduction, lines 65-68): “While the 66 exact population is unknown, it is estimated that Spain has 114.576 women sex 67 workers,[4] with one non-governmental organization (NGO) in Madrid identifying 7% of 68 TGW among the overall number of women sex workers served.[5]”

Updated sentence (Introduction, lines 67-69): “Estimates suggest there are approximately 114,576 women sex workers in Spain,(4) of whom approximately 7% are transgender women (individuals assigned male at birth who identify as women).(6,7)”

New sentence (Introduction, lines 69-71): “HIV prevalence among sex workers is estimated to be 0.8% to 2% among cisgender women (individuals assigned female at birth who identify as women),(6)…”

With regards to the request to highlighting past research to help situate the current study, we have expanded the Introduction, Methods, and Discussion to include additional context on the HIV burden, PrEP programming, and sex work in Spain, as well as data on sex work and PrEP use. With regard to evidence specific to women sex workers, our review identified 14 articles addressing aspects of the PrEP care continuum among cisgender women sex workers and 23 articles focused on transgender women sex workers. Most of these studies are now incorporated into the manuscript. During this revision, we have added references covering:

- PrEP use, changes in sexual behavior, and STI trends among transgender women sex workers (Phanuphak et al., 2018).

Introduction, lines 91-94: “To date, two prospective studies conducted in Benin and Thailand among cisgender and transgender women sex workers, respectively, have found no evidence of reduced condom use following PrEP initiation and have documented declines in STI diagnoses over the follow‑up period.(33,34)”

- Two longitudinal studies examining PrEP trajectories among cisgender and transgender women sex workers, respectively (Rao et al., 2023, Cooney et al., 2023).

Discussion, lines 447-456: “To date, only two studies have examined longitudinal PrEP trajectories among cisgender and transgender women sex workers. One longitudinal study among female sex workers in South Africa described frequent cycling on and off PrEP amongst their participants, although it did not clarify whether interruptions reflected changes in HIV risk.(51) The second, in the United States, described several distinct patterns in how transgender women stopped and restarted PrEP, One of these patterns was ‘PrEP cycling’, which tended to follow changes in HIV risk linked to shifts in socio behavioral circumstances, such as moving out of and back into sex work. Importantly, this trajectory was not associated with HIV seroconversion, suggesting that it may represent a prevention-effective adherence pattern.(52)”

- Data on the number of sex workers, and on cisgender and transgender women’s attendance at other STI clinics in Spain (Ugarte et al., 2023, Piñeiro et al., 2025)

Discussion, strengths, limitations and perspective, lines 563-565: “Moreover, attendance at this clinic was also higher than in other national cohorts,(17,64) likely reflecting the strength of the referral network.”

- Findings from the *UnPrEPared* report by European Sex Workers’ Rights Alliance, which outlines structural and social barriers that disproportionately limit cisgender women’s access to PrEP.

Discussion, strengths, limitations and perspective, lines 570-572: “Reports from European sex worker organizations describe limited PrEP awareness among cisgender women, lower perceived applicability of PrEP by healthcare providers, and continued reliance on condom‑based prevention messaging.(65)”

- Results from a previous survey conducted by our research team on PrEP awareness and perceptions among transgender women sex workers in Madrid (Vazquez et al., 2023, now available as a preprint), which was not yet published at the time of the original submission.

Discussion, strengths, limitations and perspective, lines 572-575: “In Madrid, prior surveys similarly found that a sample of predominantly street-based cisgender women sex workers had lower PrEP awareness and preferred mobile services,(13) whereas transgender women were more familiar with PrEP and favored hospital‑based clinics.(14).”

These additions strengthen the manuscript’s positioning within existing literature and clarify the specific gaps our study addresses.

**Reviewer #3: Thank you for the opportunity to review this manuscript.

I suggest major revisions are necessary to the presentation of the findings prior to a more detailed review of the manuscript.

A mixed methods approach to the data collection and analysis is commendable, but the current presentation of findings by numerous themes followed by quantitative, qualitative, and mixed methods data is cumbersome and hard to follow. The data sets can be put into conversation and simply presented as mixed methods data under themes. Additionally, I would recommend the inclusion of fewer themes, with more of an analytic (and less descriptive) focus in order to more clearly present your main findings and key messages.**

We thank the reviewer for their thoughtful and constructive feedback. In response, we have undertaken major revisions to the Results section to streamline the narrative, reduce redundancy, and more clearly highlight the novel contributions of our study.

Following the reviewer’s recommendation, we reorganized the Results section around three overarching themes: (1) PrEP initiation, (2) PrEP adherence and side effects, and (3) PrEP and sex‑work–related factors (including condom use, number of clients, and PrEP‑related communication with clients and coworkers). This new structure brings the quantitative and qualitative findings into direct conversation and avoids the previous separation into quantitative, qualitative, and mixed‑methods subsections. We also consolidated overlapping content and removed descriptive detail that did not advance the central analytic messages.

In the revised structure, embedding participant quotes within the narrative disrupted the flow of the integrated themes. For this reason, we moved all quotes to tables now included in the manuscript, where they remain accessible and clearly linked to each thematic area. This approach allowed us to maintain analytic coherence while preserving transparency and traceability of the qualitative data.

We would also like to clarify an important methodological constraint that limits our ability to conduct “further analysis” with the data. Exit interviews did not provide day‑by‑day accounts that could be directly matched to each daily survey entry. Participants reflected on patterns, motivations, and experiences across the three‑month period rather than recalling all specific events tied to individual daily reports. As a result, forcing a direct alignment between the two data sources would risk over‑interpretation. Nevertheless, the joint integration of quantitative and qualitative results at the individual level is available in the supplementary material, where readers can easily access and interpret the data collected in the study. The most relevant findings have been included in the results section of the manuscript. This approach ensures that the revised Results section reflects the highest level of integration that is methodologically appropriate while remaining faithful to the structure of the data.

We would also like to note that although the reviewer observes that some findings are already known in the broader HIV prevention literature, data specific to cisgender and transgender women sex workers remain limited. While certain patterns align with what has been reported for other populations, these results are still novel for this underrepresented group.

Finally, because the changes to the Results section are substantial, we have not reproduced them in full here and instead suggest that the reviewer compare the tracked‑changes version and the clean version of the revised manuscript. Overall, we believe these revisions substantially improve the clarity, coherence, and analytic strength of the Results section and directly address the reviewer’s concerns.

**Additional points:

Introduction:
-Would be helpful to include more information on how PrEP is delivered and accessed in Spain, as well as information about the legal status of sex work.**

We appreciate this comment. We have added further information in the Introduction regarding access to PrEP in Spain, specifying that it is a fully subsidized program, outlining the recommended dosing regimen, and noting that PrEP is primarily delivered through hospital‑based infectious diseases clinics.

Original sentence (Introduction, lines 61-63): “As part of its strategy to combat the infection, Spain recognized sex workers as a population at high risk of acquiring HIV and supported subsidized access to pre-exposure prophylaxis (PrEP) in 2019. PrEP is marketed in Spain in the form of daily pills (tenofovir and emtricitabine) and delivered in authorized healthcare centers along with other preventive measures.”

Updated sentence (Introduction, lines 60-65): “To curb the epidemic, in 2019 Spain introduced fully subsidized pre-exposure prophylaxis (PrEP) for individuals at high risk, including sex workers. PrEP is available as a daily pill (245 mg of tenofovir disoproxil fumarate and 200 mg of emtricitabine) and delivered through authorized healthcare centers, mostly hospital-based infectious diseases clinics, alongside other preventive measures.(3)”

The details regarding the criteria that women sex workers must meet to access fully subsidized PrEP through the national healthcare system have been moved from the Introduction to the Materials and Methods section, under *Study population, context, and inclusion criteria*. These criteria have also been corrected, as the original version stated that cisgender women could only access PrEP if they disclosed sex work status, which has not been the case since the 2021 revision of PrEP eligibility guidelines.

Original sentence (Introduction, lines 63-65): “Cisgender women sex workers can access PrEP if they report inconsistent condom use, while transgender women (TGW) sex workers can also qualify if they report other behaviors such as condomless anal sex or drug use with intercourse.”

New paragraph (Material and methods, Study population, context and inclusion criteria, lines 145-151): “Within the national healthcare system, women sex workers are eligible for PrEP if they disclose their sex work status and report inconsistent condom use.(12) Both transgender and cisgender women sex workers may also qualify without disclosure if they report at least two high risk behaviors in the past year, with cisgender women additionally required to report inconsistent condom use. High risk behaviors include having more than ten sexual partners, condomless anal sex, drug use associated with unprotected sex, repeated use of post exposure prophylaxis, or a diagnosis of a bacterial STI.(12)”

Additionally, we have moved the sentence regarding the legal status of sex work from the Materials and Methods section (Study population) to the Introduction.

Original sentence (Material and methods, Study population, context and inclusion criteria, lines 118-120): “While sex work itself is not illegal in Spain, it is unlawful to carry out activities such as exploitation and pimping. “

Updated sentence (Introduction, lines 66-67): “Although sex work itself is not illegal in Spain, exploitation and pimping are prohibited. “

**-As there is such a vast body of literature on PrEP, please consider adding additional references to the statements in the Introduction section.**

We appreciate this comment. In this latest revision, we have added seven new references to the Introduction, increasing the number of citations in this section from 26 to 32. Reviewer #2 also requested that we highlight past research to better situate the current study; this has been addressed as well (please see our response on pages 23–25 for a summary of the information and references added to the manuscript).

**-Line 70: delete orientation (can just say gender)**

We appreciate the correction, it has been incorporated to the manuscript:

Original sentence (Introduction, line 70): “gender orientation is not accounted for in this statistic)

Updated sentence (Introduction, line 73-74): “gender is not accounted for in this statistic”´

**-Line 76: delete ‘the’ in front of ‘PrEP’**

We appreciate the correction, it has been incorporated to the manuscript:

Original sentence (Introduction, line 76): **“**How this collective acquires knowledge and becomes motivated to start the PrEP remains poorly documented”

Updated sentence (Introduction, lines 77-78): “How women sex workers learn about PrEP and what motivates them to start PrEP remains poorly documented”

**-Consider avoiding acronyms, such as TWG**

We have removed the acronym TWG from the manuscript and instead used the term “transgender women”.

**Methods and Materials**
**-Can you further describe what an initial PrEP visit entails?**

We agree that this information is relevant. We have added the following paragraph to the manuscript to answer this question:

New paragraph (Material and Methods, Study design and data collection, lines 169-176): “The initial PrEP clinic visit routinely includes assessment of risk factors for HIV infection that qualify individuals for fully subsidized PrEP under the national health system, such as engagement in sex work; this information is subsequently submitted to the national surveillance program for PrEP use.(12) During the same visit, individuals undergo evaluation of kidney function and screening for HIV, viral hepatitis, and other sexually transmitted infections. They also receive information about the schedule of follow‑up appointments, which encompass medical evaluations, laboratory testing, and PrEP dispensation at the hospital pharmacy.”

**-How was involvement in sex work known? Especially as sex workers often do not disclose their occupation in clinical settings due to stigma.**

We appreciate the opportunity to clarify this point. Reviewer #1 had a similar question (response available in pages 6-7). Because of the referral network established through NGOs and word of mouth among colleagues, we believe sex workers attending the clinic likely felt comfortable disclosing their sex work status. However, we cannot guarantee that all sex workers revealed their status, as subsidized PrEP services could also be accessed through other reported risk factors. Information about how sex workers were identified for enrollment has been included in the methodology:

New sentence (Material and methods, Study design and data collection, lines 164-168): “The NGOs Madrid Positivo and In Género, collaborators in prior projects, informed women sex workers in their networks about the clinic and provided practical guidance on how to attend. Some participants were already familiar with PrEP and the clinic’s services through earlier fieldwork surveys assessing interest in PrEP.(13,14) Peer referral later expanded participation beyond the initial NGO network.”

New sentence (Material and Methods, Study design and data collection, lines 169-172): “ The initial PrEP clinic visit routinely includes assessment of risk factors for HIV infection that qualify individuals for fully subsidized PrEP under the national health system, such as engagement in sex work; this information is subsequently submitted to the national surveillance program for PrEP use.(12)”

New sentence (Material and Methods, Study design and data collection, lines 176-178): “In the context of this study, all women enrolled self‑identified as sex workers at the initial visit and confirmed that they had the means and time to attend the scheduled appointments.”

**-As the sample size is quite small, especially considering 3 months of enrollment, further details are required about the enrollment process.**

We appreciate this comment and have included additional information in the limitations section. Reviewer #1 had a similar question (response available in pages 5-6). Although the number of enrolled participants was small (16 women sex workers presented to the clinic during the recruitment period, of whom 15 were enrolled: 12 transgender women and 3 cisgender women), these figures are consistent with the subsequent numbers of women sex workers attending the clinic in the following months (Valencia et. al., 2024), and are higher that those of other PrEP clinics (Ugarte et al., 2023, Piñeiro et al., 2025).

During the 10 months following the onset of this study, a total of 33 transgender women, 9 cisgender women, and 18 cisgender male sex workers initiated PrEP at the study clinic (Valencia et al., 2024). This corresponds to approximately 4.2 women sex workers per month (42 women over 10 months), which is comparable to the recruitment rate observed during the study period. The cisgender‑to‑transgender sex worker ratio (0.2:1) closely mirrors the ratio observed among study participants, suggesting that our sample is representative of those presenting to this clinic.

For context, the number of women sex workers presenting to our study clinic was substantially higher than what has been reported by two other large STI clinics in Spain that have published follow‑up data on PrEP users. A PrEP cohort from an STI clinic in Barcelona (n = 297; Ugarte et al., 2023) reported that sex workers and transgender women comprised only 5% and 10% of their PrEP users between 2019 and 2020. Similarly, a cohort from the largest STI clinic in Madrid (n = 2,256; Piñeiro et al., 2025) found that transgender women represented only 0.8% of their PrEP users between 2020 and 2022. We attribute these differences to the referral network that channels a larger proportion of women sex workers to our study clinic.

This was included in the manuscript as follows:

Original paragraph (Discussion, Strength, limitations and perspectives, lines 637-640): “Firstly, the small number of participants prevents us from generalizing the results to other populations and to perform a formal comparison between cisgender and transgender women sex workers. Nevertheless, participants were enrolled in the order in which they presented to the PrEP clinic where the study was carried at. Therefore, this is a small but probably representative sample of the sex workers attending the clinic where the study took place.

Updated paragraph (Discussion, Strength, limitations and perspective, lines 557-567): “Firstly, the small number of participants limits the generalizability of our findings and precludes formal comparison between cisgender and transgender women sex workers. Nevertheless, each participant contributed a substantial amount of data (a total of 1,266 daily survey responses were collected), which strengthens the robustness of the findings. Enrollment figures were also consistent with clinic attendance patterns between 2022 and 2023 (approximately 4.2 women sex workers per month, cisgender to transgender ratio 0.2:1),(63) suggesting representativeness. Moreover, attendance at this clinic was also higher than in other national cohorts,(17,64) likely reflecting the strength of the referral network. Because of this we hypothesize that sex workers attending the clinic likely felt comfortable disclosing their status, although we cannot assume all did so, as subsidized PrEP could also be accessed through other risk factors.”

**-Consider moving some of the supplementary materials (i.e. screenshot of the app) to the main document. Can you also provide more details on the app? How was it developed? Has it been used before?**

We appreciate this comment. We have had to carefully considered how much detail to include in the main manuscript versus the supplementary materials, given the overall length of the paper.

Mpath is a digital application developed by the Faculty of Psychology and Educational Sciences at KU Leuven for mobile assessment in behavioral research. When first launched, the application was free of charge and primarily focused on tools for ecological momentary assessment (EMA), allowing researchers to collect real‑time data on participants’ experiences, behaviors, and contexts in their natural environments. Since we developed our study, Mpath has expanded to include ecological momentary intervention (EMI) tools, enabling the delivery of tailored exercises, prompts, and feedback directly to participants. In parallel, the platform has transitioned to a subscription model with tiered access for researchers, clinicians, and private sector users.

Mpath has been used in multiple research projects prior to ours, particularly in psychology and behavioral sciences, where it has supported studies on mental health, stress, and daily functioning. In our study, the application provided the opportunity to design a personalized questionnaire, deliver it directly to participants, and track response rates and answers in real time.

We have added the following information to the manuscript

Original sentence (Material and methods, study design and data collection, lines 126-139): “After lab work results for kidney function and hepatitis B virus exposure confirmed participants’ eligibility to start PrEP, they were contacted through their personal phone number and invited to download the GDPR compliant application *m-path* application on their phones,[28] through which they received brief daily self-reported questionnaires.”

Updated sentence (Material and methods, study design and data collection, lines 179-187): “After lab work results for kidney function and hepatitis B virus exposure confirmed participants’ readiness to start PrEP, they were contacted through their personal phone number and invited to download the m-path application on their phones,(43) through which they received brief daily self-reported questionnaires. M-path is a GDPR (General Data Protection Regulation) compliant application developed by the Faculty of Psychology and Educational Sciences at KU Leuven for mobile assessment in behavioral research. It was not specifically designed for this study.”

We have moved the screenshots of the application from the supplementary materials into the main manuscript. What was previously Supplementary Figure 1 (Fig S1) is now Figure 1 (Fig 1). Consequently, the numbering of the subsequent figures has been adjusted accordingly.

**-The Medication Adherence Model needs to be described within the text.**

We appreciate this comment. While the Medication Adherence Model (MAM) was only briefly referenced in the Materials and Methods section, we have now expanded its description in the Introduction to provide clearer conceptual grounding

New sentences (Introduction, lines 103-113); “Originally developed to guide clinicians in evaluating adherence among patients with hypertension, the Medication Adherence Model (MAM) provides a useful framework for understanding how individuals make and sustain medication taking decisions.(36) Through its three core concepts (Purposeful Action, Patterned Behavior and Feedback), the model provides a structured lens for examining what motivates individuals to take or skip medication, how daily patterns form over time, and how these patterns interact with broader structural and occupational contexts such as sex work. Moreover, MAM’s distinction between intentional nonadherence (e.g., cycling off during perceived low risk periods) and unintentional nonadherence (e.g., forgetting doses or facing access barriers) is particularly relevant for providers, as it highlights different points of intervention to support adherence. Guided by this framework,…”

**Results
-Table 1 can be presented as aggregate data and not per participant.**

Because this study was designed as a series of case studies with mixed‑methods assessment, part of the analysis necessarily occurs at the individual level. We therefore consider it important to retain Table 1 in its current format, as it highlights individual differences that are central to the study’s contribution and complements more traditional group‑level analyses. While certain identifiers have been removed or generalized (e.g., country of origin replaced with “Latin America”) to protect participant privacy, the table still conveys meaningful contextual information about each participant without compromising confidentiality.

**-Line 270-1: “When considering only the days with answers to the daily survey, adherence oscillated between 77.7% to 100% (95% in average for both groups) (S1 Table).” I am not clear on why you would or could exclude days without responses – wouldn’t a lack of response be anticipated to be linked with lower adherence?**

We agree with this comment, and the sentence in question (Results, PrEP adherence, Quantitative findings, lines 269-270) has been removed from the manuscript to avoid misinterpretation.

**-The findings section is very long. It is not clear what the Mixed Methods section is adding. Instead, the quantitative and qualitative findings need to be put into conversation and thematically presented together. Additional supplementary tables might not be needed if key findings are presented in the body of the manuscript. Much of the data presented in the findings is already known and well-documented in the literature. I would encourage the authors to focus in on which data points are novel. Further analysis can be done as many of the themes can be lumped together and presented with more analytic detail.**

We thank the reviewer for their thoughtful and constructive feedback. In response, we have undertaken major revisions to the Results section to streamline the narrative, reduce redundancy, and more clearly highlight the novel contributions of our study.

Following the reviewer’s recommendation, we reorganized the Results section around three overarching themes: (1) PrEP initiation, (2) PrEP adherence and side effects, and (3) PrEP and sex‑work–related factors (including condom use, number of clients, and PrEP‑related communication with clients and coworkers). This new structure brings the quantitative and qualitative findings into direct conversation and avoids the previous separation into quantitative, qualitative, and mixed‑methods subsections. We also consolidated overlapping content and removed descriptive detail that did not advance the central analytic messages.

In the revised structure, embedding participant quotes within the narrative disrupted the flow of the integrated themes. For this reason, we moved all quotes to tables now included in the manuscript, where they remain accessible and clearly linked to each thematic area. This approach allowed us to maintain analytic coherence while preserving transparency and traceability of the qualitative data.

We would also like to clarify an important methodological constraint that limits our ability to conduct “further analysis” with the data. Exit interviews did not provide day‑by‑day accounts that could be directly matched to each daily survey entry. Participants reflected on patterns, motivations, and experiences across the three‑month period rather than recalling all specific events tied to individual daily reports. As a result, forcing a direct alignment between the two data sources would risk over‑interpretation. Nevertheless, the joint integration of quantitative and qualitative results at the individual level is available in the supplementary material (S2-S5 Table), where readers can easily access and interpret the data collected in the study. The most relevant findings have been included in the results section of the manuscript. This approach ensures that the revised Results section reflects the highest level of integration that is methodologically appropriate while remaining faithful to the structure of the data.

We would also like to note that although the reviewer observes that some findings are already known in the broader HIV prevention literature, data specific to cisgender and transgender women sex workers remain limited. While certain patterns align with what has been reported for other populations, these results are still novel for this underrepresented group.

Finally, because the changes to the Results section are substantial, we have not reproduced them in full here and instead suggest that the reviewer compare the tracked‑changes version and the clean version of the revised manuscript. Overall, we believe these revisions substantially improve the clarity, coherence, and analytic strength of the Results section and directly address the reviewer’s concerns.

**-I would not include a subsection with just two cisgender women sex workers.**

We appreciate the reviewer’s comment regarding the subsection on cisgender women sex workers. Our initial intention in including this subsection was to ensure transparency. Although only two cisgender women completed the study, their quantitative and qualitative findings were consistent, and we felt that briefly presenting them together provided useful context without implying formal comparative analysis.

However, we understand the reviewer’s concern about dedicating a standalone subsection to such a small subgroup. In response, we have removed this subsection from the main text. We have added the following sentence to the results section to keep part of the information:

New sentence (Results, prep use and sex-work related factors, lines XX): “Notably, the cisgender women in our study chose not to disclose their PrEP use to any clients.”

Information on average reported PrEP adherence, side effects, condom use and number of clients is also retained in the manuscript at the beginning of each subsection. All specific information about the cisgender women participants remains available in the supplementary materials (S2-S5 Tables), ensuring that readers can still access these data without interrupting the flow of the Results section.

**-Please note that I am not able to assess the heat maps or models.**

Thank you for noting this. The submitted manuscript did not include legends for each study figure, although this information was embedded within the figures themselves. In case this led to difficulties interpreting the visuals, we have now added legends to all figures. These legends explicitly define the X and Y axes and specify the color coding where appropriate, ensuring that readers can accurately interpret the visual data. Below are the updated legends:

**Fig 2. Heatmap representation of PrEP adherence and side effects during the study period.** The Y axis represents individual participants, and the X axis represents days since study initiation. Color coding: blue indicates days when PrEP was taken, orange indicates days when PrEP was not taken, and gray denotes missing data. Dots mark days on which a side effect was reported.

**Fig 3. Heatmap representation of condom use with clients during the study period.** The Y axis represents individual participants, the X axis represents days since study initiation. Color coding: blue indicates days when a condom was used in all sexual encounters or when no clients were reported; orange indicates days when condom use occurred in less than 100% of sexual encounters; and gray denotes missing responses.

**Fig 4.** **Time series representing the number of clients for each individual participant during the study period.** Each panel represents an individual participant, labeled from 1 to 14. The X axis indicates days since study initiation, and the Y axis shows the number of clients reported per day (range: 0–8).

**Fig 5. Study results mapped to the Medication Adherence Model.** This figure organizes participant-reported experiences into three domains of the Medication Adherence Model: Purposeful Action (A), Patterned Behavior (B), and Feedback (C).

Additionally, to ensure full transparency, the data and code used to generate the heat maps and models are openly available (https://osf.io/6rdxh/), allowing complete reproducibility of our results. Several co‑authors on the manuscript also have extensive experience with quantitative analyses of individual time series and oversaw the development and validation of these components. We hope this provides confidence in the robustness of the analytic approach.

**Discussion
-Subheadings do not need to be repeated in the discussion.**

We appreciate this comment. Given the length of the manuscript and the range of topics addressed in the Discussion, we thought that having subheadings would improve readability and help guide the reader through the different themes. Nevertheless, we have removed them following recommendations from the reviewer.

**-Line 569 – I would avoid saying sex work negatively impacts PrEP adherence, but rather shift work and changing schedules/routines.**

We appreciate this suggestion and have modified the phrasing introducing the paragraph as follows:

Original sentence (Discussion, PrEP adherence and side effects, lines 569-570): “Moreover, our study supports that sex work negatively impacts PrEP adherence[11] and provides insight on the different ways that it does so.”

Updated sentence (Discussion, PrEP adherence and side effects, lines 487-488): “Moreover, our study provides insight on the different ways in which sex work might complicate consistent PrEP adherence.(17).”

The paragraph that follows then offers more detailed information about irregular shift work, changing schedules, and other work‑related factors.

**-Line 573-4: “In addition, the use of alcohol and drugs with clients impacted PrEP intake, sometimes because it made them more forgetful, but mostly because participants opt to skip PrEP to avoid unintended side effects of mixing” – was this presented in the data?**

We understand this comment. This issue is mentioned in the Results, but we did not specify how many participants were affected. In the interviews, four participants reported difficulties with PrEP adherence in the context of alcohol or drug use. One participant (ID7) attributed missed doses to forgetfulness, while three others (ID3, ID8, and ID11) described intentionally skipping PrEP to avoid potential side effects when mixing it with substances. Additionally, one participant (ID4) reported avoiding PrEP while taking an antibiotic for the same reason.

Original sentence (Results, PrEP adherence, Qualitative findings, lines 311-312): “Additionally, some participants would consciously opt to skip their PrEP dose to avoid mixing it with alcohol or drugs.”

Updated sentence (Results, PrEP adherence and side effects, lines 331-332): “Recreational drug use was more commonly associated with intentional nonadherence than with forgetfulness.”

**-Line 583-4: “We hypothesize that stigma or forgetfulness prevented them from sharing these concerns with their prescribing physicians.”- is there anything in the data pointing to forgetfulness related to inquiring about medication interactions? Discussion statements should link to what was presented in the findings section (and not in the supplementary tables).**

Thank you for highlighting this point. We acknowledge that the statement was not grounded in our study findings but instead reflected our own hypothesis to explain why some participants had unanswered questions about drug–drug interactions, which in turn led them to stop PrEP to avoid potential side effects from mixing substances. These questions may have arisen after clinic appointments, or reflect discomfort discussing PrEP use with certain providers. However, because these interpretations are not directly supported by the data presented in the findings, we have removed this sentence to ensure that the Discussion remains closely aligned with the evidence reported in the manuscript.

**-Line 595-7: “The report of the cisgender women in our study is in concordance with the lack of risk compensating behaviors identified in the only study on this area carried amongst female sex workers in Benin.[41]” – I believe many PrEP studies have also looked at condom use. This statement should be clarified.**

Our review of scientific publications and conference presentations identified 14 articles addressing aspects of the PrEP care continuum among cisgender women sex workers and 23 among transgender women sex workers. Within this body of work, only two studies specifically examined condom use, number of sexual partners, or STI outcomes following PrEP initiation. A study conducted in Benin (2020) among cisgender women sex workers reported stable condom use, supported by both self‑report and biomarkers of semen exposure, alongside a decline in STI prevalence from 15.8% to 2.1% over 24 months of follow‑up. Similarly, a study in Thailand (2016–2017) including transgender women sex workers found no increase in self‑reported number of sexual partners, condomless sex, or drug use during 12 months of follow‑up, and documented a reduction in syphilis cases at 6 and 12 months compared to baseline.

We have added information about the Thailand study (Phanuphak et al., 2018) not previously included in the manuscript in the Introduction:

Original sentence (Introduction, lines 90–93): “The evidence for risk‑compensating behaviors among cisgender and TGW sex workers is scant, with just one prospective PrEP observational study in this topic carried out in Benin that failed to show evidence of risk compensation among female sex workers after two years of follow‑up.[17]”

Updated sentence (Introduction, lines 91-94): “To date, two prospective studies conducted in Benin and Thailand among cisgender and transgender women sex workers, respectively, have found no evidence of reduced condom use following PrEP initiation and have documented declines in STI diagnoses over the follow‑up period.[17] (REF)”

Given that these are the only studies we identified that assess behavioral or STI outcomes after PrEP initiation among women sex workers, we believe the original statement remains accurate. Nevertheless, acknowledging the possibility that additional relevant evidence may exist despite our thorough search, we have revised the sentence in the Discussion to ensure clarity and to avoid implying that only one study exists:

Original sentence (Discussion, Condom use and number of clients, lines 595-597): “The report of the cisgender women in our study is in concordance with the lack of risk compensating behaviors identified in the only study on this area carried amongst female sex workers in Benin.[41]”

Updated sentence (Discussion, Condom use and number of clients, lines XX): “The report of the three cisgender women in our study aligned with prior studies that show no evidence of decreased condom use after PrEP initiation among women sex workers.(33,34)”

**Limitations
-I am not sure that you can call your sample representative.**

We appreciate the opportunity to clarify this point (please see also our answer to Reviewer #1 on pages 5-7 and to Reviewer #3 on pages 29 -31). To clarify, we assume representativeness of total number of women sex workers attending the PrEP clinic where the study took place based on the findings exposed in the following paragraphs, not the larger population of women sex workers in Madrid.

Because of the referral network established through NGOs and word of mouth among colleagues, we believe sex workers attending the clinic likely felt comfortable disclosing their sex work status. A marker of this is that our PrEP clinic had a larger number of sex workers attending the clinic than what has been reported by two other large STI clinics in Spain that have published follow‑up data on PrEP users (Valencia et al., 2024, Ugarte et al., 2023, Piñeiro et al., 2025). However, we cannot guarantee that all sex workers revealed their status, as subsidized PrEP services could also be accessed through other reported risk factors.

During the 10 months following the onset of this study, a total of 33 transgender women, 9 cisgender women, and 18 cisgender male sex workers initiated PrEP at the study clinic (Valencia et al ., 2024). This corresponds to approximately 4.2 women sex workers per month (42 women over 10 months), which is comparable to the recruitment rate observed during the study period. The cisgender‑to‑transgender sex worker ratio (0.2:1) closely mirrors the ratio observed among study participants, suggesting that our sample is representative of those presenting to the PrEP clinic where the study took place.

For context, a PrEP cohort from an STI clinic in Barcelona (n = 297; Ugarte et al., 2023) reported that sex workers and transgender women comprised only 5% and 10% of their PrEP users between 2019 and 2020. Similarly, a cohort from the largest STI clinic in Madrid (n = 2,256; Piñeiro et al., 2025) found that transgender women represented only 0.8% of their PrEP users between 2020 and 2022. We attribute these differences to the referral network that channels a larger proportion of women sex workers to our study clinic.

This information was included in the manuscript as follows:

Original paragraph (Discussion, Strength, limitations and perspectives, lines 637-640): “Firstly, the small number of participants prevents us from generalizing the results to other populations and to perform a formal comparison between cisgender and transgender women sex workers. Nevertheless, participants were enrolled in the order in which they presented to the PrEP clinic where the study was carried at. Therefore, this is a small but probably representative sample of the sex workers attending the clinic where the study took place.

Updated paragraph (Discussion, Strength, limitations and perspective, lines 557-567): “Firstly, the small number of participants limits the generalizability of our findings and precludes formal comparison between cisgender and transgender women sex workers. Nevertheless, each participant contributed a substantial amount of data (a total of 1,266 daily survey responses were collected), which strengthens the robustness of the findings. Enrollment figures were also consistent with clinic attendance patterns between 2022 and 2023 (approximately 4.2 women sex workers per month, cisgender to transgender ratio 0.2:1),(63) suggesting representativeness. Moreover, attendance at this clinic was also higher than in other national cohorts,(17,64) likely reflecting the strength of the referral network. Because of this we hypothesize that sex workers attending the clinic likely felt comfortable disclosing their status, although we cannot assume all did so, as subsidized PrEP could also be accessed through other risk factors.”

**-Limitations require more discussion of the enrolment process, small sample size, and need for disclosure.**

We appreciate this comment. First, we want to highlight that more information about enrollment process and need for disclosure of sex work has been added to the Material and Methods of the manuscript.

New sentence (Material and methods, Study design and data collection lines 164-168): “The NGOs Madrid Positivo and In Género, collaborators in prior projects, informed women sex workers in their networks about the clinic and provided practical guidance on how to attend. Some participants were already familiar with PrEP and the clinic’s services through earlier fieldwork surveys assessing interest in PrEP.(13,14) Peer referral later expanded participation beyond the initial NGO network.”

New sentence (Material and Methods, Study design and data collection, lines 169-170): “The initial PrEP clinic visit routinely includes assessment of risk factors for HIV infection that qualify individuals for fully subsidized PrEP under the national health system, such as engagement in sex work; this information is subsequently submitted to the national surveillance program for PrEP use.(12)”

New sentence (Material and Methods, Study design and data collection, lines 176-178): “In the context of this study, all women enrolled self‑identified as sex workers at the initial visit and confirmed that they had the means and time to attend the scheduled appointments.”

Because of the referral network established through NGOs and word of mouth among colleagues, we believe sex workers attending the clinic likely felt comfortable disclosing their sex work status. A marker of this is that our PrEP clinic had a larger number of sex workers attending the clinic than what has been reported by two other large STI clinics in Spain that have published follow‑up data on PrEP users. However, we cannot guarantee that all sex workers revealed their status, as subsidized PrEP services could also be accessed through other reported risk factors.

During the 10 months following the onset of this study, a total of 33 transgender women, 9 cisgender women, and 18 cisgender male sex workers initiated PrEP at the study clinic.(Valencia et al., 2024) This corresponds to approximately 4.2 women sex workers per month (42 women over 10 months), which is comparable to the recruitment rate observed during the study period. The cisgender‑to‑transgender sex worker ratio (0.2:1) closely mirrors the ratio observed among study participants, suggesting that our sample is representative of those presenting to the PrEP clinic where the study took place.

For context, a PrEP cohort from an STI clinic in Barcelona (n = 297; Ugarte et al., 2023) reported that sex workers and transgender women comprised only 5% and 10% of their PrEP users between 2019 and 2020. Similarly, a cohort from the largest STI clinic in Madrid (n = 2,256; Piñeiro et al., 2025) found that transgender women represented only 0.8% of their PrEP users between 2020 and 2022. We attribute these differences to the referral network that channels a larger proportion of women sex workers to our study clinic.

The limitations and strengths of the study sample and enrollment process have been included in the discussion of the manuscript as follows:

Original paragraph (Discussion, Strength, limitations and perspectives, lines 637-640): “Firstly, the small number of participants prevents us from generalizing the results to other populations and to perform a formal comparison between cisgender and transgender women sex workers. Nevertheless, participants were enrolled in the order in which they presented to the PrEP clinic where the study was carried at. Therefore, this is a small but probably representative sample of the sex workers attending the clinic where the study took place.

Updated paragraph (Discussion, Strength, limitations and perspective, lines 557-567): “Firstly, the small number of participants limits the generalizability of our findings and precludes formal comparison between cisgender and transgender women sex workers. Nevertheless, each participant contributed a substantial amount of data (a total of 1,266 daily survey responses were collected), which strengthens the robustness of the findings. Enrollment figures were also consistent with clinic attendance patterns between 2022 and 2023 (approximately 4.2 women sex workers per month, cisgender to transgender ratio 0.2:1),(63) suggesting representativeness. Moreover, attendance at this clinic was also higher than in other national cohorts,(17,64) likely reflecting the strength of the referral network. Because of this we hypothesize that sex workers attending the clinic likely felt comfortable disclosing their status, although we cannot assume all did so, as subsidized PrEP could also be accessed through other risk factors.”

**-“The fact that cisgender women sex workers’ need to disclose sex work status to qualify for PrEP, along with lack of awareness, may explain the disproportionate ratio between transgender and cisgender women sex workers in the study” -but transgender women would also need to disclose sex work.**

Thank you for pointing this out. We have corrected the statement, as it was an error. The initial national PrEP guidelines (2019) included men who have sex with men and transgender women with several risk factors, as well as women sex workers who reported inconsistent condom use. In this context, transgender women could access PrEP without disclosing sex work status, whereas cisgender women were required to do so. The guidelines were subsequently modified in 2021 to also include cisgender women with several risk factors other than sex work. Details about the eligibility criteria for PrEP has been expanded and moved from the Introduction to Material and Methods.

Original sentence (Introduction, lines 63-65): “Cisgender women sex workers can access PrEP if they report inconsistent condom use, while transgender women (TGW) sex workers can also qualify if they report other behaviors such as condomless anal sex or drug use with intercourse.”

New paragraph (Material and methods, Study population, context and inclusion criteria, lines 145-151): “Within the national healthcare system, women sex workers are eligible for PrEP if they disclose their sex work status and report inconsistent condom use.(12) Both transgender and cisgender women sex workers may also qualify without disclosure if they report at least two high risk behaviors in the past year, with cisgender women additionally required to report inconsistent condom use. High risk behaviors include having more than ten sexual partners, condomless anal sex, drug use associated with unprotected sex, repeated use of post exposure prophylaxis, or a diagnosis of a bacterial STI.(12).”

Nevertheless, broader contextual factors may also help explain the disproportionate ratio between cisgender and transgender women sex workers enrolled in our study. The *UnPrEPared* report by the European Alliance of Sex Workers’ Rights highlights that cisgender women in several European countries (Spain was not included) face additional barriers to PrEP access, including national guidelines that prioritize other at‑risk groups, limited awareness among both women and healthcare providers, and provider pressure to rely on condoms for HIV prevention and contraception. In Madrid, two prior cross‑sectional surveys of women sex workers similarly found that although interest in PrEP was high across groups, cisgender women had lower awareness and preferred mobile clinics (importantly this sample had an over representation of street-based sex workers), whereas transgender women were more familiar with PrEP and favored hospital‑based infectious diseases clinics. This preference, combined with their higher HIV risk, may have contributed to higher number of transgender women sex workers attending the clinic where the study took place.

Therefore, the sentence highlighted by Reviewer #3 has been deleted from the manuscript, and we have added a new paragraph explaining the disproportionate ratio between cisgender and transgender women sex workers.

Original sentence (Discussion, Strengths, limitations and perspectives, lines 642-644): “The fact that cisgender women sex workers’ need to disclose sex work status to qualify for PrEP, along with lack of awareness, may explain the disproportionate ratio between transgender and cisgender women sex workers in the study”

Updated paragraph

New paragraph (Discussion, Strengths, limitations and perspectives, lines 568-581): “Secondly, the disproportionate ratio of cisgender to transgender women sex workers enrolled may reflect a combination of structural and contextual factors. Reports from European sex worker organizations describe limited PrEP awareness among cisgender women, lower perceived applicability of PrEP by healthcare providers, and continued reliance on condom‑based prevention messaging.(65) In Madrid, prior surveys similarly found that a sample of predominantly street-based cisgender women sex workers had lower PrEP awareness and preferred mobile services,(13) whereas transgender women were more familiar with PrEP and favored hospital based clinics.(14) These differences in awareness, service preferences, and perceived HIV risk may have contributed to the higher representation of transgender women in our study. Nevertheless, the purpose of this innovative mixed methods study was not to stablish a comparison between groups, but to highlight each individual’s PrEP journey, an information that complements that obtained in studies using traditional, group-level only results, and brings us closer to clinical practice..”

**-Conclusion can be shortened and the Figure should not be in the conclusion section. It’s also not clear how the Medication Adherence Model was applied in the study – this should have been central to the presentation of findings.**

We appreciate this comment. We have shortened the Conclusions section by removing the following sentences:

Conclusions (lines 669-675): “… among other reasons, participants were afraid of acquiring other sexually transmitted infections. PrEP communication was often limited to a close circle of friends (often sex workers) who offered support by sharing knowledge, and reminders to take the pill. However, this information was not always true or accurate, highlighting the need for accessible official channels where participants could verify information. Importantly, two participants became open “advocates” for PrEP in their community. Otherwise…”

Conclusions (lines 676-679): “ Finally, strict national regulations around PrEP dispensation at designated hospital pharmacies represented an additional layer of difficulty for study participants to obtain their refills on time. Study results mapped to the Medication Adherence Model can be found in Fig 4.”

In addition, we have moved the reference to Fig 4 from the Conclusions to the Results section, and its label was updated to Fig. 5 to reflect the new enumeration.:

Original sentence (conclusions, lines 678-679): “Study results mapped to the Medication Adherence Model can be found in Fig 4.”

New location of the sentence (results, Prep use and related work factors, lines 424): “Study results mapped to the Medication Adherence Model can be found in Fig 5”

We have expanded the Introduction and Methods to provide clearer information about the Medication Adherence Model and how it was applied in the study:

New sentences (Introduction, lines 103-113): “ Originally developed to guide clinicians in evaluating adherence among patients with hypertension, the Medication Adherence Model (MAM) provides a useful framework for understanding how individuals make and sustain medication taking decisions.(36) Through its three core concepts (Purposeful Action, Patterned Behavior and Feedback), the model provides a structured lens for examining what motivates individuals to take or skip medication, how daily patterns form over time, and how these patterns interact with broader structural and occupational contexts such as sex work. Moreover, MAM’s distinction between intentional nonadherence (e.g., cycling off during perceived low risk periods) and unintentional nonadherence (e.g., forgetting doses or facing access barriers) is particularly relevant for providers, as it highlights different points of intervention to support adherence. Guided by this framework,”

Updated sentence (Materials and methods, Qualitative measures, lines 228-232): “The MAM served as a theoretical framework.(36) The guide explored motivations behind PrEP initiation and sustained adherence, the development of daily routines, and the impact of PrEP on work life and vice versa, with special attention to number of clients, condom use, drug use, stigma, and communication with clients and other sex workers.”

Updated sentence (Material and methods, quantitative measures, lines 198-204): “PrEP adherence, side effects, number of clients, and condom use were measured every day with a single item for each variable detailed below (screenshots of the app-based questionnaire are shown in Fig 1). We selected these variables based on their relevance to the study outcome (understanding the phenomenon of PrEP adherence and its bidirectional relationship with sex work), and their suitability for daily measurement, which allowed us to capture short term fluctuations and day to day patterns in participants’ experiences.”

**Reviewer #4: Yes from my quick search this has not been published before. One article was published on determinants of intention to use PrEP (**[**https://link.springer.com/article/10.1007/s10508-024-02834-4**](https://link.springer.com/article/10.1007/s10508-024-02834-4)**). See my feedback/comments below for minor revisions.**

**Additional Comments
- In introduction, would change per "100.000 habitants to 100,000" same for "114.576" in Introduction**

We appreciate the reviewer highlighting this mistake. The use of a dot as a thousands separator reflects Spanish formatting conventions. We have corrected the numbers in the introduction (e.g., “100.000 habitants” changed to “100,000” and “114.576” changed to “114,576”) to align with English conventions.

**- Line 61-62 would specify dosing at least once of tenofovir disoproxil fumarate/emtricitabine** **300/200 mg po daily and/or other regimens as appropriate)**

We appreciate this comment. The dosing has been specified in the manuscript as follows:

Original sentence (Introduction, lines 61-63): “PrEP is marketed in Spain in the form of daily pills (tenofovir and emtricitabine)”

Updates sentence (Introduction, lines 62-63): “PrEP is available as a daily pill (245 mg of tenofovir disoproxil fumarate and 200 mg of emtricitabine)…”

**- Line 91-93 can you clarify what the Benin paper showed, not clear**

We appreciate this comment. We have clarified what we meant by “no evidence of risk‑compensating behaviors” and have added a second relevant study to the statement.

Original sentence (Introduction, line 91-93): “The evidence for risk compensating behaviors among cisgender and TGW sex workers is scant, with just one prospective PrEP observational study in this topic carried out in Benin that failed to show evidence of risk compensation among female sex workers after two years of follow up.[17]”

Updated sentence (Introduction, lines 91-94): “To date, two prospective studies conducted in Benin and Thailand among cisgender and transgender women sex workers, respectively, have found no evidence of reduced condom use following PrEP initiation and have documented declines in STI diagnoses over the follow up period.(33,34)”

**- Line 116 methods would clarify that you mean does not have chronic hep B infection**

Thank you for catching this. While being hepatitis B virus (HBV) positive is not a contraindication for initiating PrEP, national protocols require that individuals with HBV be evaluated by a specialist. In cases of chronic hepatitis B infection, patients must also be counselled about the importance of strict adherence to PrEP to avoid hepatitis flares if treatment is interrupted. For the purposes of this study, we therefore aimed to enroll participants without evidence of hepatitis B infection.

Original sentence (Methods, study population and inclusion criteria, line 116): “…(5) meeting medical criteria to start PrEP (normal kidney function and negative hepatitis B status or vaccinated),…”

Updated sentence (Methods, study population and inclusion criteria, lines 155-157)“…(5) meeting medical criteria to start PrEP immediately (normal kidney function and no medical conditions requiring additional monitoring, such as chronic hepatitis B infection);…”

**- Line 128-129 what is GDPR-compliant m-path application (wording also to be checked -- you say application twice in sentence)**

We appreciate this comment. The suggested changes have been incorporated to the manuscript as follows:

Original sentence (Methods, Study design and data collection, lines 128-129)**:** “…GDPR-compliant application *m-path* application…”

Updated sentence (Methods, Study design and data collection, line 185): “…GDPR (General Data Protection Regulation) compliant application…”

**- Results: Demographics line 209 change to "approached for recruitment for the study."**

We appreciate this suggestion, it has been incorporated as follows:

Original sentence (Results, Demographics, lines 209): “A total of 16 women sex workers were approached.”

Updated sentence (Results, Demographics, lines 274-275): “A total of 16 women sex workers were approached for recruitment for the study”

**- Line 240 spelling of penis to be corrected**

Thank you for the correction. This sentence was removed from the manuscript during the latest revision suggested by Reviewer #3

Original sentence (Results, PrEP initiation, line 240): “… certain sexual positions in which the woman can not visualize the pennis.”

**- Line 267 can you report the PreP adherence by which participant (ID 1 through 15) ie which participant had adherence of 37% and which had 100% (if you can report)**

We appreciate this comment. The participant‑level adherence data (IDs 1–15) are already available in supplementary material S1 Table. We believe that reproducing these values within the main manuscript may be cumbersome and could disrupt the flow of the Results section. However, we would be happy to move S1 Table into the main text if the reviewer considers it essential.

**- Can shorten the discussion where you resummarize the results (would synthesize more tightly)**

We appreciate this recommendation, the first paragraph of the discussion has been shorten as follows:

Original paragraph (Discussion, lines 524-538): “We enrolled 12 transgender and 3 cisgender women sex workers, all immigrants from Latin America, in an intensive longitudinal mixed methods study to capture their experience taking PrEP during the first three months of therapy, with special emphasis in understanding the dynamics of PrEP adherence. Quantitative results showed that average reported PrEP adherence was above 70%, with missed doses being randomly distributed, and that condom use and number of clients did not significantly change during the study period. Qualitative results showed that the main reason to start PrEP was concern about condom rupture and/or removal by clients. Facilitators of PrEP adherence included personal motivation, creation of daily routines, and use of personal alarms. Barriers to PrEP adherence included side effects from PrEP and its coingestion with other substances, and changes in daily routine related to work, and travel. Strict prescription protocols represented an additional layer of difficulty. Most participants relaxed their use of condoms with few selected clients based on “loyalty”, high economic reward or personal attraction. PrEP and HIV related stigma were common among the sex worker community.”

New paragraph (Discussion, lines 429-438): We enrolled 12 transgender and 3 cisgender women sex workers, all immigrants from Latin America, in an intensive longitudinal mixed methods study to capture their experience during the first three months of PrEP use. Average reported PrEP adherence was above 70%. Adherence was supported by personal motivation, daily routines, and the use of alarms. Missed doses were randomly distributed and reflected both unintentional non adherence (side effects, work-related disruptions) and intentional non adherence (avoiding side effects that could affect work performance). Most participants relaxed their use of condoms with few selected clients based on “loyalty”, high economic reward or personal attraction, although this did not appear to affect the overall number of clients. PrEP and HIV related stigma were common among the sex worker community.

**- Figure 2 clarify x axis**

Figure 2 (now renamed as Figure 3, see below) presents a heatmap of PrEP adherence and reported side effects over the study period. The Y axis displays individual participants, ordered from highest to lowest number of daily questionnaires completed. The X axis represents the number of days since each participant’s initiation into the study. While the intended follow‑up period was 90 days, some participants continued completing daily questionnaires for an additional 3 to 4 days beyond the three‑month mark. These responses were retained in the dataset, which explains why the figure does not form a perfect square.

To improve clarity, we have now included a legend for each study figure mentioned in the manuscript. These legends explicitly define the X and Y axes and specify the color coding used.

**Fig 3. Heatmap representation of condom use with clients during the study period.** The Y axis represents individual participants, the X axis represents days since study initiation. Color coding: blue indicates days when a condom was used in all sexual encounters or when no clients were reported; orange indicates days when condom use occurred in less than 100% of sexual encounters; and gray denotes missing responses.

**- Overall can shorten # of quotes in body of text since you have quotes in the supplementary files**

We appreciate the reviewer’s suggestion to reduce the number of quotes included in the body of the text. In response to this comment, and in alignment with the broader restructuring of the Results section prompted by Reviewer #3, we have removed all quotations from the main text. To maintain transparency and ensure that readers still have full access to the qualitative material, all quotes are now presented in tables that are included within the main manuscript rather than in the supplementary materials.
